# Componential modeling of argumentative essay writing from multiple online sources: a Bayesian network approach

**DOI:** 10.3389/fpsyg.2025.1560088

**Published:** 2025-05-15

**Authors:** Anisha Singh, Yuting Sun, Patricia A. Alexander, Hongyang Zhao

**Affiliations:** ^1^Department of Psychology, San Francisco State University, San Francisco, CA, United States; ^2^Department of Teaching, Learning & Curriculum, University of North Florida, Jacksonville, FL, United States; ^3^Department of Human Development and Quantitative Methodology, University of Maryland, College Park, College Park, MD, United States; ^4^Department of Educational Psychology, University of Illinois at Urbana-Champaign, Champaign, IL, United States

**Keywords:** argumentation, multiple source use, argumentative essay writing, Bayesian network analysis, multiple documents, college students

## Abstract

**Introduction:**

Writing argumentative essays using multiple sources is a critical skill for college students, yet it remains a significant challenge. Despite previous research acknowledging this difficulty, the specific dynamics of the argumentative essay writing process and where breakdowns occur remain unclear.

**Methods:**

College students wrote argumentative essays on a controversial topic after reading multiple documents. The data were fitted to two competing theory-based Bayesian networks, a method highly suited to the modeling of cognitive processes identified with argumentative writing.

**Results:**

The best-fitting model showed that the argumentative essay task is both initiated and sustained by higher-order integration components. This model lends support to the description of the process of argumentation writing from multiple documents put forth by the stage-based Integrated Framework of Multiple Texts. Further, we found that the process of argumentation falters due to students' inability to frame counterarguments and their non-optimal critical analysis.

**Discussion:**

This research not only enriches our understanding of the mechanics of argumentative writing from multiple sources, but the innovative Bayesian approach could lead to further refinement of the model by future researchers.

## Introduction

The importance of oral or written argumentation is well-established in the literature. Educational research has demonstrated that the ability to formulate cogent arguments is critical to learning across domains (Asterhan and Schwarz, 2007; De La Paz, 2005; Wiley and Voss, 1999). Further, with its emphasis on evidence and consideration of varying and contradictory perspectives, argumentation is at the heart of a democratic education (Gutmann, 1999; Hess and Avery, 2008). It should come as no surprise, therefore, that this manner of thinking and reasoning has been the subject of philosophical and psychological analysis since the time of Aristotle. In his most famous work on the topic of oral argumentation written in the 4^th^ century BCE, *The Art of Rhetoric*, Aristotle drew on the history of ancient logic and intricately analyzed the art of persuasion. *The Art of Rhetoric* is the foundational treatise on which modern argumentation theory is based (Aristotle, 4th century BCE, 2019; Perelman and Olbrechts-Tyteca, 1969; van Eemeren, 2013).

The importance of argumentation is further evidenced in the attention it is paid in educational policies and practices intended to promote learner development and the overall wellbeing of society (Jiménez-Aleixandre and Erduran, 2007; Asterhan and Schwarz, 2016). For example, the K−12 Common Core State Standards for writing lists the ability to “write arguments to support claims in an analysis of substantive topics or texts using valid reasoning and relevant and sufficient evidence” as a curricular goal (National Governors Association Center for Best Practices and Council of Chief State School Officers, 2010, p. 18). Despite these standards and policy mandates, far too many students struggle with argumentation, as numerous research studies have documented (e.g., Kuhn, 1991; McCann, 1989; Means and Voss, 1996). Researchers have demonstrated that students are unable to recognize and apply argumentative text structures (Chambliss and Murphy, 2002; Freedman and Pringle, 1984); have difficulty providing appropriate evidence to justify claims or positions (Kuhn and Modrek, 2021; List et al., 2022); and fail to offer counterarguments or rebuttals (Ferretti and Fan, 2016; Leitão, 2003; Mason and Scirica, 2006).

Such academic challenges are amplified when argumentation takes the form of a written product (National Assessment of Educational Progress, 1994, 1999, 2002). This is because skilled writing is in itself a complex activity depending on cognitive, contextual, and motivational factors (Bereiter and Scardamalia, 1987; Galbraith and Torrance, 1998; Graham, 2018). Thus, producing a solid piece of argumentation in writing entails the transformation of knowledge and requires skills underlying composition, cognizance of the constraints imposed by the argumentative genre, specific topic, and the audience being addressed, while being driven and efficacious at managing the challenges of the writing task.

An additional source of difficulty in constructing arguments arises from the use of multiple texts, particularly with online resources (Alexander and the Disciplined Reading and Learning Research Laboratory, 2012; Stadtler, 2017). Those challenges pertain to the proliferation of information available online, multiple perspectives on the same issue, and varying degrees of source credibility and content accuracy (Braasch et al., 2014; McGrew, 2021). Consequently, students called upon to craft argumentative essays from multiple documents must be able to evaluate and integrate the information from multiple documents, even before they begin to write. Cognizant of these challenges, researchers have turned their attention on examining and promoting competencies related to using multiple sources. These competencies include the ability to assess the reliability of sources and establish connections among ideas across different documents (Anmarkrud et al., 2013; Braasch and Bråten, 2017; Britt and Rouet, 2012). Developing these skills is crucial for crafting argumentative essays within the pluralistic information landscape of the internet.

The current study builds upon and extends the aforementioned research on argumentation, particularly in its written form within the context of using multiple sources. It employs an innovative statistical method—Bayesian Network analysis—to model the componential processes involved in producing a quality argumentative essay from multiple documents.

Specifically, in this study, students were required to access, read, and integrate information from a library of online documents that varied in both source and content credibility. We selected only original documents from the internet to create the library. We carefully curated documents that represented various combinations of source and content credibility. For instance, we included documents from credible sources that were found to contain content of questionable credibility. This is different from previous studies where only the credibility of sources, and not the content of those sources, was manipulated (e.g., Ecker and Antonio, 2021; Sparks and Rapp, 2011; van Boekel et al., 2017). Further, we employed Bayesian Network analysis, a probability-based technique, that allows for the modeling of causal relations among components and make predictions about the relative importance of each component to the production of a quality argumentative essay. The rationale for the use of this more novel technique was to shed light on the complex interrelations among the components that constitute the argumentative writing process. This analysis would also allow us to identify components of argumentation that seem particularly challenging for students.

Due to the fact that we used Bayesian Network analysis as a theoretically driven approach where key components were specified prior to modeling, we first discuss argumentation and the components entailed in its execution. We then describe the particular framework of multiple source use into which the writing of an argumentative essay was embedded. Finally, given the somewhat novel modeling procedure we apply, we briefly overview Bayesian Network analysis.

## The process of argumentation

Argumentation is a complex process that has been studied across multiple disciplines, each offering unique perspectives and models. While philosophical approaches often emphasize logic, resulting in the well-known inductive and deductive argument structures, this study adopts a broader, dialectical view of argumentation (van Eemeren and Grootendorst, 2004). This choice is motivated by our focus on everyday contexts involving controversial social topics, rather than purely scientific or philosophical debates.

In the dialectical approach, van Eemeren et al. (1996, p. 5) define an argument as “a verbal and social activity of reason aimed at increasing (or decreasing) the acceptability of a controversial standpoint for a listener or reader, by putting forward a constellation of propositions intended to justify (or refute) the standpoint before a ‘rational judge'.” This definition emphasizes argumentation as a communicative, rational activity aimed at influencing standpoints through justification and refutation of anticipated counterarguments.

The dialectical view frames argumentation as a goal-directed, interactional process where two or more parties engage to resolve a conflict of opinion. An argument comprises a claim that is supported by evidence, anticipates potential challenges, and is strengthened by addressing counterarguments (Walton, 2007).

Given the cognitive focus of our study, we aim to elucidate the process of argumentation from a cognitive perspective, rather than examining the textual structure of arguments produced by our students. We posit that the generation of claims, the provision of justifications, and the formulation of counterarguments are cognitive processes that manifest as observable features in the resulting text. This psychological approach conceptualizes argumentation not merely as a social and verbal activity, but critically as a series of complex mental operations.

Our framework posits that the cognitive processes underlying argumentation—specifically, claim formulation, evidence evaluation, and anticipation of opposing viewpoints—are reflected in both the structural and content-based elements of the argumentative text. We hypothesize that these cognitive operations leave discernible traces in the textual output, providing a window into the mental processes of the arguer (Galbraith, 1998; Van Wijk, 1998).

This cognitive-centric model allows us to investigate the intricate interplay between internal cognitive mechanisms and their external manifestations in argumentative discourse. By focusing on these cognitive underpinnings, we aim to develop a more nuanced understanding of the mental operations that drive effective argumentation, potentially offering insights into cognitive strategies that can enhance argumentative skills.

## Integrated framework of multiple texts

The process of argument construction based on multiple texts has been a focus in the body of literature on multiple source use (Barzilai et al., 2021; De La Paz and Felton, 2010; Vandermeulen et al., 2020). In multiple source use (MSU) tasks that culminate in the production of argumentation, students need to read multiple texts on the focal issue and integrate information and perspectives from different documents to build their own arguments. The complex processes of using multiple texts to produce a desired outcome have been characterized by several theoretical frameworks (e.g., Documents Model Framework, Perfetti et al., 1999; Internet Information Problem-Solving model, Brand-Gruwel et al., 2009; Multiple Documents-Task-based Relevance Assessment and Content Extraction model; Rouet, 2006). Common to these models is the emphasis on how learners consolidate information within and across multiple documents to create an integrated representation of the texts and the topic. Among the various models, we turned to the Integrated Framework of Multiple Texts (IF-MT, List and Alexander, 2019) as a guide for our effort to understand the process of integrating information from multiple texts in producing quality argumentation.

**Figure 1:**
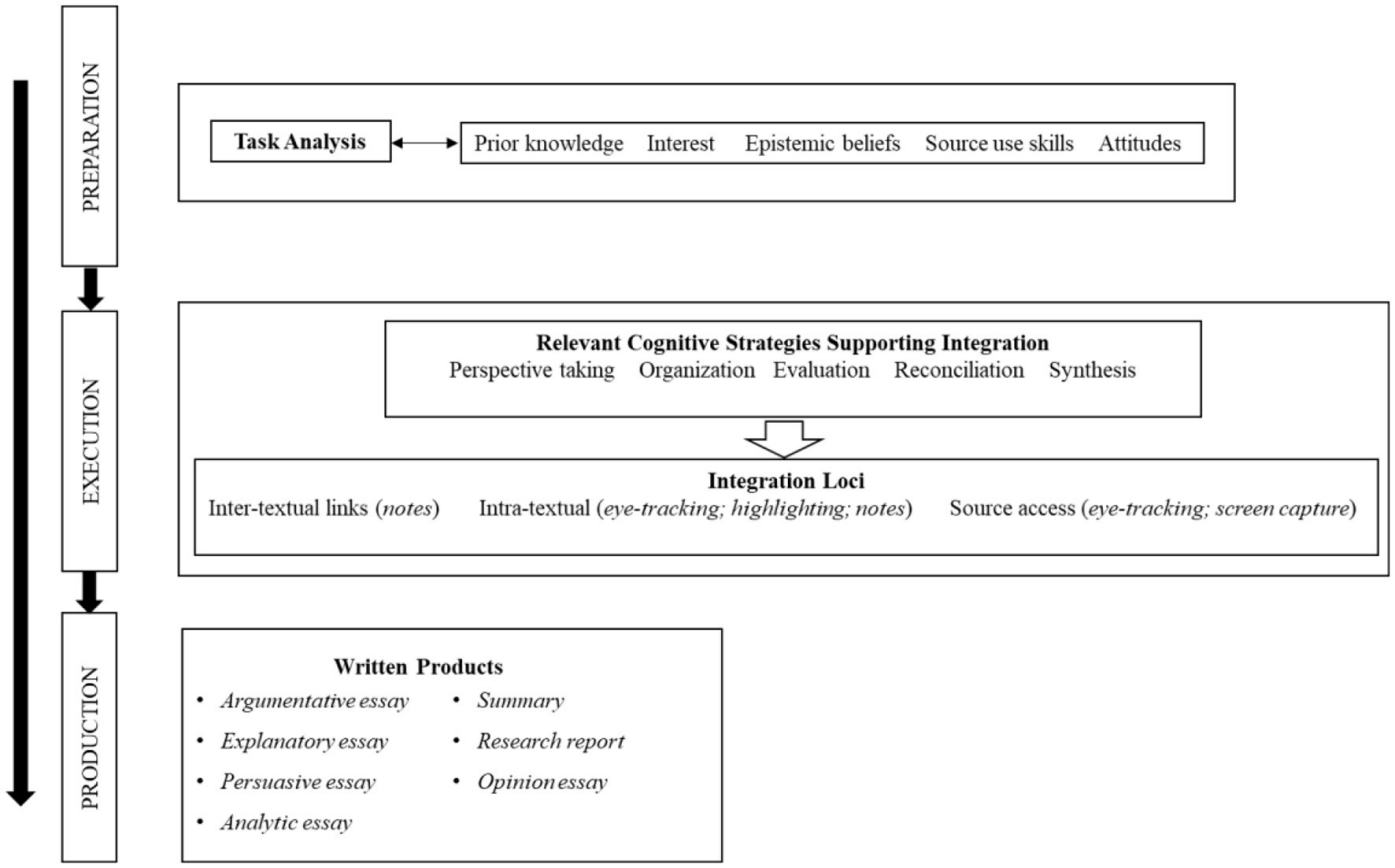
Section of the integrated framework of multiple texts (IF-MT) focusing on written products and the processes underlying task analysis and integration. The model shows the multi-stage process of MSU writing. Italics represent manifestations or traces of processes that researchers can directly access.

The IF-MT is a comprehensive framework that was a consolidation of other existing models. The framework delineates three stages that explain the complex process underpinning students' multiple source use—preparation, execution, and production. The unfolding of argumentative writing in the MSU contexts in IF-MT's three stages is visually depicted in Figure 1.

In the first stage, *preparation*, students orient themselves by conducting task analysis to determine the requirements of the assignment at hand and begin mentally mapping the steps toward completion. Students' analysis of the task is influenced by the interplay of their individual characteristics (e.g., knowledge, interest, attitude) and external task demands. For example, students' prior knowledge about what argumentation constitutes can influence the quality of their argumentative essays (Nussbaum, 2011).

The second stage, *execution*, is where students implement the steps for completing the task, and the stances they adopted begin to manifest in external actions. During this stage, students search, navigate, select, and read sources and then forge associations within and across documents. Through these complex processes, students engaged in an argumentative task may develop mental representations of the informational terrain of the topic by integrating diverse perspectives and supporting justifications from various documents, which may shape or reshape their stances on the topic.

The final phase of the IF-MT is the production stage, wherein students engage in the complex process of text generation. In the context of our study, this culminated in the creation of argumentative essays. Crucially, the mental representations formed during the preceding planning and execution stages significantly influence the production process. We chose to focus on this stage where text production happens because it provides a window into cognitive processes (Galbraith and Torrance, 1998).

Unlike traditional writing models (Hayes and Flower, 1980), the IF-MT posits that production is not merely a mechanical conversion of thought to text, but rather a dynamic, on-line process that plays a pivotal role in shaping the final product. This aligns with the text-production perspective, which conceptualizes the act of writing itself as a process that engenders new understanding and facilitates “knowledge transformation” (Bereiter and Scardamalia, 1987; Galbraith, 1998).

This theoretical stance suggests that the production stage offers a unique opportunity to assess the efficacy of students' comprehension of task parameters and their level of information integration. As Galbraith (1998) argues, analyzing the text as a window into cognitive processes can be a particularly fruitful approach, as it centers the intricate processes involved in text production.

In educational research and practice, written products—particularly argumentative essays—are frequently employed in multiple source use tasks (Luna et al., 2022; Mateos et al., 2018). Recognizing this, our study focused on the production stage, leveraging the IF-MT to elucidate the cognitive components that contribute to the composition of high-quality argumentative essays derived from multiple documents.

This approach allows us to examine the cognitive processes involved in text production through an analysis of their traces in the final product. By doing so, we aim to contribute to a more nuanced understanding of the complex interplay between task and integration components that underpin the creation of an argumentative essay for the broader purposes of influencing writing instruction and support.

## Identifying core components of multiple-text-based argumentation

Based on the extensive literature on argumentation and multiple source use just overviewed, we identified core components of argumentative essay writing involving multiple documents. The components included both essential elements for completing the argumentative essay task (i.e., task parameters) and core processes for achieving integration of multiple sources (i.e., integration components). Each of the task parameters and the integration components captures complex cognitive processes that are manifested in and inferred from the written product.

### Task parameters

Drawing on the pragma-dialectical approach to argumentation (van Eemeren and Grootendorst, 2004), we delineated the following task parameters: (1) presenting a *claim*, (2) providing *justifications* for the claim, (3) addressing *counterarguments* through rebuttal or refutation, (4) using multiple sources. The first three parameters (claim, justifications, and counterarguments) directly reflect the dialectical nature of argumentation. The fourth parameter, using multiple sources, was deemed necessary given the multiple-text-based nature of the argumentative essay task. This parameter adds an additional layer of complexity to the task, requiring students to navigate and select multiple documents.

Further, foundational to generating a written product was students' *writing ability*. In effect, the ability to communicate through writing was judged as foundational to the production of an argumentative essay.

These delineated task components emerge from the cognitive processes underlying writer's representation of the rhetorical problem (Galbraith, 1998; Flower and Hayes, 1980). This cognitive perspective allowed us to conceptualize the task parameters as manifestations of the mental operations involved in writing an argumentative essay.

### Integration components

*Integration*, as defined by Alexander and the Disciplined Reading and Learning Research Laboratory (2020) is “the meaningful consolidation of elements found within and across information sources that results from the *analysis* and *synthesis* of their contents” (p. 408). This definition highlights the foundational roles of analysis and synthesis to integration. These processes can occur throughout the three stages described in the IF-MT (List and Alexander, 2019).

At the preparation stage, students may engage in preliminary task analysis as they inspect task requirements, available materials, and contextual characteristics vis-à-vis their knowledge, beliefs, and motivations. This initial assessment results in the adoption of a default stance toward task completion (e.g., critical analytic; List and Alexander, 2017). The chosen stance influences enactment of the task parameters that are reflected in the final written product.

In execution, analysis can occur when students critically evaluate the quality of the sources and their contents and identify the relations between pieces of information within and across documents. As students process the texts, they may synthesize the contents across texts depending on the consistent or conflicting nature of the information being synthesized. The depth and quality of this analysis and synthesis manifest in the sophistication of the argumentative essay.

The production stage is where earlier cognitive processes along with the cognitive processes associated with writing become externalized in the written essays. Effective integration in writing requires coherent expression of ideas, with content-based connections between sentences and paragraphs. As Alexander and the Disciplined Reading and Learning Research Laboratory (2020) explain, “cohesion requires not only the synthesis of content across information sources, but also the analytical ability to produce inferences that bridge the informational gaps that will inevitably exist” (p. 411).

This conceptualization of cohesion adopts a cognitive perspective, aligning with the mental processes fundamental to problem-solving. Creating a coherent text necessitates that the writer maintains a goal-directed approach throughout composition. This cognitive lens emphasizes that coherence is not merely a textual feature, but rather the result of deliberate mental operations initiated in the preparation and execution stages, and externalized in the production stage.

Given the importance of these cognitive processes, we focused on three core integration components for producing an integrated written essay: (1) *critical analysis*, (2) *synthesis*, (3) content-based *overall cohesion*.

### Unpacking the interplay between task parameters and integration components

Together, the enactment of the task parameters and integration components undergird the production of argumentative essays based on multiple sources. What needs to be further explored is how these components work together in the production of the written essay. We claim that the process of composing an argumentative essay unfolds in a way that certain components are prerequisites for the manifestation of other specified components. For example, in an argumentative essay, a claim must be forwarded before providing justifications or addressing potential counterarguments. The directionality of this particular process is straightforward—justification follows a claim—but the association between some of the other components is less established. For example, is critical analysis a prerequisite for synthesis or vice versa? Does critical analysis come into play before a student forms a counterargument? Therefore, in this study, we explored the specific linkages among the identified task and integration components using Bayesian network analysis. Before describing the models we tested, we present a brief description of the Bayesian networks used to formulate those models and make inferences about the processes entailed in argumentative essay writing.

## Bayesian network analysis

Bayesian network analysis is a powerful statistical tool that allows for the modeling of complex causal relations among variables. At its core, a Bayesian network is a graphical model that represents probabilistic relationships among a set of variables (Jensen, 1996; Jensen and Nielsen, 2007). It consists of two key components: structure and strength. The structure is represented by a Directed Acyclic Graph (DAG), where variables are depicted as nodes and the dependencies between them as arrows (Pearl and Russell, 2003; Murphy, 1998). The strength of these relations is quantified by conditional probability distributions representing how strongly variables in the network influences one another.

In our study of argumentative writing, we employed a hybrid approach to Bayesian network analysis. We specified the network structure *a priori*, while the parameters (strengths of relationships) were learned from data, integrating theory with computational learning. This approach of combining expert knowledge and machine learning is particularly suitable for modeling complex cognitive processes.

**Figure 2:**
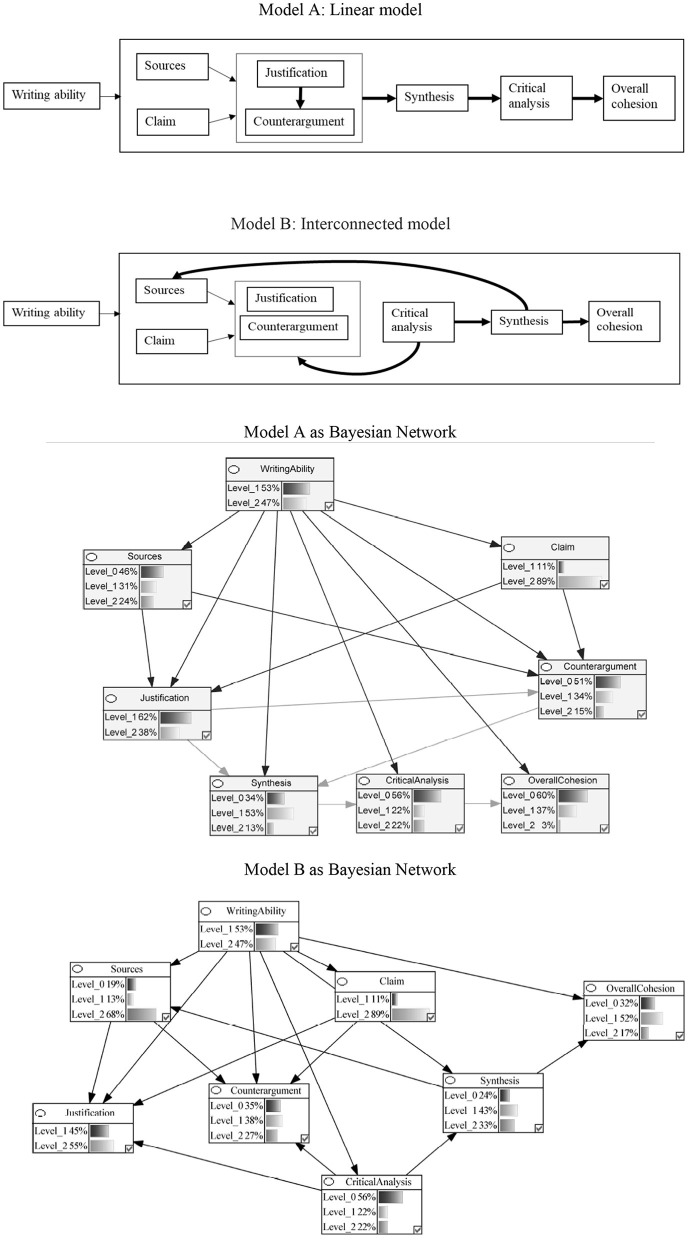
Theoretical Models and Corresponding Bayesian Networks of the Componential Process of MSU Argumentative Essay Writing in the Production Stage. The percentages represent the probability of the undergraduates performing at a specific level in this study. Arrows represent causal relations between variables. Level_0 = Below Average; Level_1 = Average; Level_2 = Above Average.

Our models represented various components of argumentative writing as nodes in the network, with arrows indicating the hypothesized causal relationships between these components. Figure 2 provides a visual representation of these network, illustrating how we conceptualized the process of argumentative essay writing as a series of interconnected cognitive components that leave traces in the text product.

Bayesian network analysis offers several advantages over frequentist statistical techniques. For example, a key advantage of Bayesian networks over generalized linear models is their ability to compute the impact of changes in a subset of variables that are part of an entire network or a subset of it (Pearl and Russell, 2003). In our case, we can investigate how selection of sources and synthesis of content may impact the quality of justifications, counterarguments, and overall cohesion of the essay. Bayesian network analysis also offers several advantages over traditional structural equation modeling (SEM) approaches for modeling argumentative essay writing. Specific to our study, it provides a robust method for handling ordinal data with a small sample size, while capturing complex, non-linear relations between components.

Most importantly, Bayesian networks offer a distinct advantage in result interpretation, particularly for argumentative essay writing. Unlike the continuous estimates in SEM, Bayesian networks express outcomes as probabilities of achieving specific score levels. This approach aligns more closely with how educators conceptualize student performance, making findings more intuitive and actionable. For example, a Bayesian network can directly convey the probability of a student achieving a high score on the *claim* component based on their *source* use performance. This probabilistic framework captures the nuanced relationships between components more effectively than linear estimates, better reflecting the complex nature of argumentative writing with multiple documents.

Building upon these advantages, our study leveraged Bayesian updating to further enhance our analysis (Almond et al., 2015; Pearl, 1988). Bayesian updating applies Bayes' theorem to the complex interdependencies within the network, allowing us to refine our understanding of the argumentative writing process as new data are considered. In practice, Bayesian updating involves computing the posterior probability of an event given its prior probability and likelihood function. With our model structure specified based on theory and parameters determined from empirical data, we used this updating process to draw nuanced inferences about argumentative essay writing.

This approach transcends simple correlation analysis, enabling us to explore probabilistic causal relationships (Pearl, 2000). When we update a node—meaning we set its value to a specific state—the Bayesian network recalculates probabilities throughout the system. This updating process reveals how new information about one variable propagates to influence both its parent nodes (variables that directly affect it) and child nodes (variables it directly affects), creating ripple effects of probability changes throughout the entire network. This dynamic probability provides a comprehensive view of the interrelationships within the argumentative writing process.

For instance, we could examine how improvements in source selection might cascade through the network, affecting justification quality and overall essay cohesion. This capability provides actionable insights for educators, highlighting which components of argumentative writing are most challenging for students and how interventions in one area might impact performance in others.

### Model diagnostics

To validate our Bayesian network models, we employed cross-validation, one of several diagnostic methods available for this purpose (Sinharay, 2006). While other techniques such as item fit plots, item-test statistics, and posterior predictive model checking exist, cross-validation is particularly well-suited for assessing a model's predictive power (Sinharay, 2006; Yan et al., 2003).

Specifically, we utilized leave-one-out cross-validation, an extension of the k-fold cross-validation technique, to estimate our models' goodness of fit. This method involves training the Bayesian network n times, where n equals our sample size. In each iteration, the algorithm excludes one data point, uses the remaining data to train the model, and then predicts the excluded point. This process is repeated for all n data points, providing a robust assessment of the model's predictive accuracy across our entire dataset.

This approach allowed us to evaluate how well our models predicted students' performances in argumentative writing tasks, ensuring the reliability and generalizability of our findings. For a more detailed description of our Bayesian network analysis methodology, please refer to the Supplementary material.

## Research questions and hypotheses

Students' struggle with argumentative writing has been well-documented and empirically explored. However, challenges with argumentation get amplified when students function in contexts with multiple documents. Despite an abundance of theoretical models of the argumentative process, there is limited understanding of how this componential process unfolds in a multiple source task. Further, we do not know where the process of argumentative writing breaks down for most students. Therefore, in this study, we posed the following research questions:

(1) *Based on Bayesian network analysis, which of the plausible theoretical models best captures the process of writing an argumentative essay from multiple documents? Specifically, which model - linear or interconnected - better predicts students' performance on task and integration components?*

Based on the IF-MT, we hypothesized that understanding the task requirements is a prerequisite to producing an argumentative essay. The task components we included in our model were: (a) the linguistic ability to write effectively (*writing ability*), without which the student is unable to initiate the entire process of producing an argumentative essay; (b) using *sources*; (c) stating a *claim*; (d) providing *justifications*; and (e) discussing *counterarguments*. The sequential interconnections were ascertained based on how the argumentative writing unfolds. The next set of building blocks were the three theoretically determined integration components relevant to MSU written tasks: (a) *critical analysis* of the sources and contents, (b) *synthesis* of the multiple sources and the content encountered within and across documents, and (c) *overall cohesion* of the ideas presented in the written product. According to the IF-MT, critical analysis and synthesis occur in the execution stage and are evidenced in the written outcomes in the production stage when readers make intra- and inter-textual links. Although the sequence in which the task components unfold is somewhat apparent, the precise manner in which the integration components play out in the writing process needs to be investigated.

In this study, we examined two plausible models that varied in their interconnections among components: a linear model and an interconnected model. Figure 2 presents the conceptual models depicting these plausible interrelations, which were subsequently converted into Bayesian networks for analysis.

It is crucial to note that each component in our models represents a complex cognitive process, each worthy of individual study and computationally complex to model. The underlying cognitive processes are likely distributed in nature (McClelland et al., 1986). For instance, *synthesizing* ideas relies on distributed semantic memory (Galbraith, 1998). However, our focus in this study is not on the internal workings of each component, but rather on the orchestration of the components in text production.

Our emphasis on this level of analysis stems from two key considerations. First, we aim to describe cognitive processes at a level that can lead to actionable educational implications. Second, while each component and their orchestration involve complex cognitive processes, the product of each component is traceable in the essay text. Thus, we view the text as a window into how these coarser-grained components come together in the writing process.

This approach allows us to examine the architecture of the written product as a reflection of the sequential interaction of these components. By focusing on this level of analysis, we seek to bridge the gap between complex cognitive processes and observable outcomes in argumentative writing, potentially informing educational practices and interventions.

(2) *What does Bayesian updating indicate about the relative importance of the components to the writing of the argumentative essay?*
  (2a) *How do early task components, particularly writing ability, formulating a claim, and source use, influence subsequent task and integration components in the argumentative writing process?*
  (2b) *What is the impact of critical analysis on other task and integration components throughout the network?*
  (2c) *How does synthesis ability affect both preceding and subsequent components in the argumentative text production?*

Using the selected Bayesian network, we estimated the probabilities associated with the sequential and bi-directional causal interrelations between the components. Specifically, we tested a series of different performance scenarios with the Bayesian network. For example, we modeled a scenario in the Bayesian network where a student exhibited the highest level of critical analysis and observed how the other interrelated components changed. Given the interconnected nature of the Bayesian network, this was akin to asking: what level of performance does a student need on task and integration components to exhibit the highest level of critical analysis in their essay? We conducted this analysis, known *as belief updating*, to determine which components were most critical for the process of argumentative essay writing.

For argumentative essay writing, we hypothesized that writing ability would have a substantial influence on all subsequent task and integration components. We expected that formulating a clear claim early in the writing process would positively impact justifications, counterarguments, and integration components. Furthermore, given that this is a multiple source use task, we predicted that effective source use would be a key component for producing a quality essay, influencing both task components (justifications and counterarguments) and integration components.

Regarding critical analysis, we hypothesized that it would show strong effects on both task and integration components (e.g., improving source selection, enhancing synthesis and justifications). This is because critical analysis operates at multiple levels—evaluating source credibility, content verification, assessing the logical connection between claims and their supporting evidence.

For synthesis ability, we hypothesized that it will have a significant impact on justifications, counterarguments, and overall cohesion. We anticipated that strong synthesis skills will be reflected in improved integration of multiple sources in the essay, contributing to a more coherent and well-supported argumentative essay.

Concerning counterarguments, we predicted that including them will be crucial for the integration process, particularly enhancing critical analysis and synthesis. We expected that strong performance in counterarguments will positively influence essay cohesion. The pivotal role of counterarguments was anticipated due to their critical importance in dialectical argumentation, where they serve to strengthen the overall argument by addressing potential objections and alternative viewpoints (Nussbaum and Schraw, 2007; Walton, 2007).

Overall, we expected that the interplay between task components (writing ability, claim formulation, source use, justifications, and counterarguments) and integration components (critical analysis, synthesis, and cohesion) will be complex and multidirectional. We anticipated that improvements in one area will potentially influence both preceding and subsequent components in the argumentative writing process, reflecting the interconnected nature of cognitive processes involved in writing argumentative essays from multiple sources.

## Method

### Participants

Participants in this study were 95 undergraduate students at a large Mid-Atlantic university (57 females, 1 non-binary, 1 preferred not to say). These students were enrolled in a general education course aimed at developing their learning capabilities through discussions of relevant topics (e.g., problem-solving, transfer, reasoning, and motivation) and practical learning experiences. Students represented varied majors, including arts and humanities (e.g., English, anthropology, philosophy), social sciences (e.g., criminal justice, economics, psychology), natural sciences (e.g., mathematics, physics, biology), and applied sciences (e.g., engineering, computer science, information science). The participants included freshmen (20%), sophomores (25.3%), juniors (26.3%), and seniors (28.4%), with a mean age of 20.19 (*SD* = 1.47). Their racial backgrounds were diverse, with 42.1% self-identified as White, 20% as Asian, 13.7% as Black, 4.2% as Latino, 15.8% as multiracial, and 4.2% as other races.

The study was approved by the Institutional Review board.

### Multiple source use task

Students completed the multiple source use task as an assignment in the course. The task required students to read texts in a provided digital library and write an argumentative essay regarding the claim statement: “*Students today are overly dependent on technology to the detriment of their social, physical, emotional, and academic well-being*.” Specifically, students were required to select at least four out of the ten documents in the library and use what they read to compose an argumentative essay. This MSU task is an integral component of the course, as challenges of discerning credible sources and using online information for learning were key topics in the course. Students were not provided any pre-task instruction about how to read or use multiple texts to construct argumentative essays. Rather, their performance was used as a basis for post-task discussion on the challenges of engaging in such multiple document tasks, which are relatively common for college students.

The topic for the MSU task was chosen for its perceived controversy and interestingness, as reported by students (*n* = 48) enrolled in the same course in the previous semester. Those students represented similar demographic and academic backgrounds as the participants in the current study. Among a list of ten topics, the question about students' overdependence on technology was rated as the most controversial (*M* = 57.1, *SD* = 24.6) and most interesting (*M* = 68.5, *SD* = 23.0) on a scale of 0 to 100.

#### Digital library

**Figure 3:**
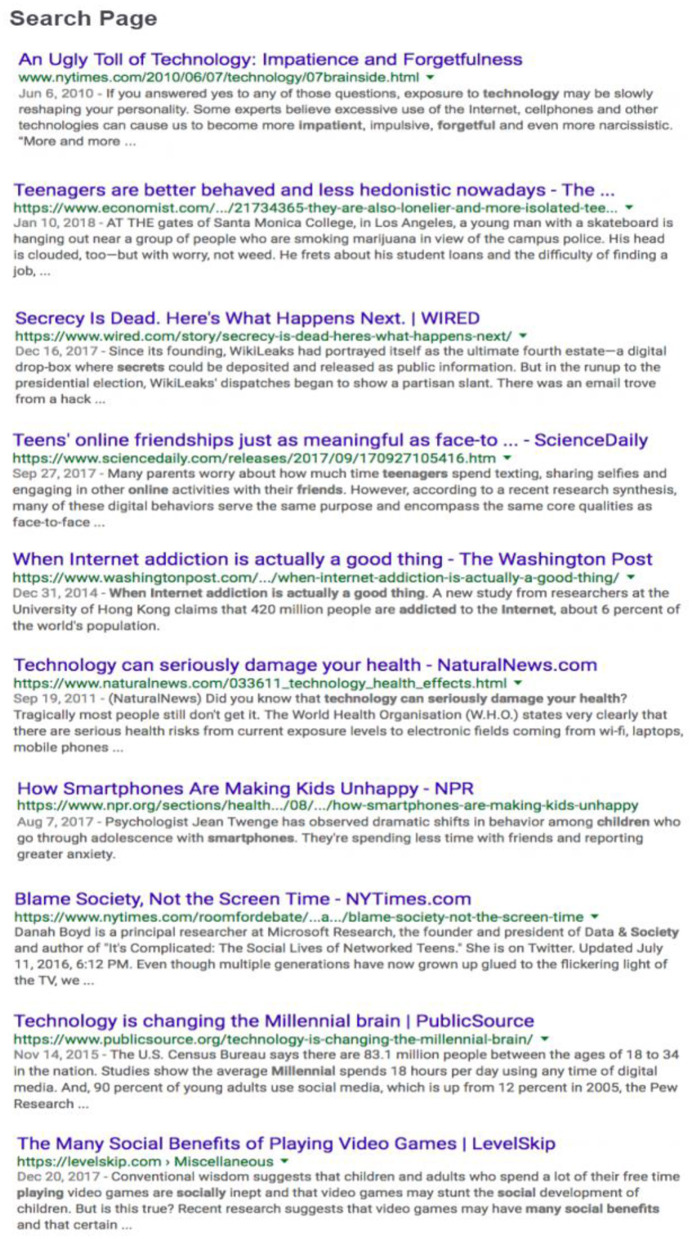
Digital library designed to resemble a Google search page.

The 10-document digital library was linked to a menu that resembled a Google search page with the title, publisher, date of publication, URL, and a blurb for each document (see Figure 3). The documents linked to the menu were screenshots of original websites from the Internet with minimal modifications (e.g., removing the comment section). The documents varied by type (e.g., blog post, newspaper article, popular magazine), source credibility, content trustworthiness, and perspectives on the topic (see Table 1). Text length varied from 340 to 1,787 words. To verify the features of the documents in the digital library, the first and fourth authors independently coded each for the level of source credibility (high or low), overall content trustworthiness (high or low), and topic stance (agree, disagree, or neutral). The interrater agreement was 96.7%. Consensus was reached on all these dimensions of text features through discussion.

**Table 1:** Details of the documents in the digital library.

**Title**	**Type**	**Source**	**Year**	**Length**	Topic position^*^	**Source credibility**	**Content credibility**
An Ugly Toll of Technology: Impatience and Forgetfulness	Newspaper	*New York Times*	2010	859	Agree	High	High
Teenagers are Better Behaved and Less Hedonistic Nowadays	Newspaper	*The Economist*	2017	1787	Neutral	High	High
Secrecy is Dead. Here's What Happens Next.	Magazine	*Wired*	2017	1235	Agree	High	Low
Teens' Online Friendship Just as Meaningful as Face-to-Face Ones	Research press release	*Science Daily*	2017	340	Disagree	High	High
When Internet Addiction is Actually a Good Thing	Newspaper	*Washington Post*	2014	863	Disagree	High	Low
Technology Can Seriously Damage Your Health	Blog	*Natural News*	2011	631	Agree	Low	Low
How Smartphones Are Making Kids Unhappy	Website (Radio station)	*NPR*	2017	723	Agree	High	Low
Blame Society, Not the Screen Time	Newspaper	*New York Times*	2016	655	Disagree	High	Low
Technology is Changing the Millennial Brain	Blog	*Public Source*	2015	1438	Agree	Low	Low
The Many Social Benefits of Playing Video Games	Blog	*Levelskip*	2017	1269	Disagree	Low	High

^*^Topic position refers to the position presented in the document vis-à-vis the controversial statement: “Students today are overly dependent on technology to the detriment of their social,

physical, emotional, and academic well-being.”

Sources judged as high in credibility were from reputable publishers or websites known for accurate and reliable reporting of information (e.g., the New York Times and The Economist). Sources low in credibility were from less well-established or personal outlets (e.g., PublicSource and Levelskip), and sites known for propagating pseudoscientific information (e.g., Natural News). Among the ten documents in the library, seven were judged as high in source credibility and three were rated as low in credibility.

As for content trustworthiness, the content of documents was considered trustworthy if the author presented relevant, accurate, and objective evidence to support their claim, and if the evidence was communicated in a logical and rigorous manner. Four documents were in the high content credibility category. In contrast, documents were considered to present low-credibility content if the authors made vague or unsupported arguments, presented claims without citations, or based the claims on personal experience and questionable evidence. Six documents were classified as low in content credibility.

Finally, with regard to the stances represented in the documents, four focused on the harmful effects of technology. For example, the article by NPR “How Smartphones are Making Kids Unhappy,” argued for the deleterious impact of smartphones on children's socioemotional wellbeing. Five documents forwarded a positive view of technology. For example, the Washington Post article, “When Internet Addiction is Actually a Good Thing,” viewed high Internet addiction rates as a sign of socioeconomic improvements. One document, “Teenagers are Better Behaved and Less Hedonistic Nowadays” published by The Economist, showed a neutral stance by presenting evidence that supported both positive and negative sides of technology.

#### Procedure

The students completed the MSU task independently on their laptops. The task consisted of four components: (a) pre-reading questions, (b) digital library reading, (c) post-reading questions, and (d) argumentative essay writing. The first three components were completed on Qualtrics^®^, while the essays were composed in Microsoft^®^ Word. First, students provided consent to participate in the study and completed the pre-reading questions about their demographic information and their initial positions on the controversial topic. Specifically, students were presented with the topic statement and were asked to indicate the extent to which they agreed or disagreed with that statement on a 0 to 100 scale (0 = strongly disagree, 100 = strongly agree). They were then directed to record their position on the controversial topic and to provide a brief justification for that position.

Next, students proceeded to navigate the digital library and select documents for reading. Students were instructed to scan the search result page and read the linked documents. They were required to select at least four documents to read in depth. As they read the selected documents, they could highlight any information that stood out to them as particularly trustworthy or questionable and rate the overall credibility and usefulness of each document at the end of the page on scales of 0 to 100. The highlighting and document ratings, which were not part of this analysis, were elements of a separate project investigating students' source and content evaluations (Sun et al., 2020). Students were also allowed to take notes while they read.

After reading, students responded to the post-reading questions about their final positions on the topic. For these questions, they were again presented with the topic statement and were asked to indicate whether or not their position had changed and the extent to which they now agreed or disagreed with the statement on a 0–100 scale.

Finally, students composed their argumentative essays in a Word document. They were presented with the claim statement and were asked to follow the stated directions:

*Clearly* ***state*** *your position and write an argumentative essay regarding the viewpoint presented above*. ***Explain*** *and* ***justify*** *your position with sound reasoning*.

The students could refer to their notes as they wrote but could not re-access the digital library. They were instructed to write as much as they needed to articulate their arguments, but no specific length requirements were given.

Students were given instructions about each component of the MSU task in class and completed the task as an assignment outside of class. They were told to find a quiet place to first complete the Qualtrics^®^ portion of the task (i.e., pre-reading questions, research in digital library, and post-reading questions) in one sitting and then write the essay as required. There were no time constraints on their reading, writing, or question responses, and all task components were completed within a 5-day period. For this study, we focused only on the argumentative essays for a Bayesian network analysis.

## Argumentative essay scoring

The argumentative essays were scored based on a researcher-developed rubric that consisted of two sets of parameters: (a) adherence to task requirements, which included five key parameters for a multiple-source-based argumentative essay task (i.e., stating a *claim*, presenting *justifications*, referencing multiple *sources*, discussing *counterargument*s, and demonstrating adequate *writing ability*); and (b) integration of multiple sources, which consisted of three core components for producing a well-integrated written essay (i.e., *critical analysis* of sources and contents, *synthesis* of multiple documents, and *overall cohesion* of information presentation). Each component was scored on a 0 to 2 scale, with a total possible score ranging from 0 to 16 (see Table 2 for rubric details).

**Table 2:** Rubric for scoring the argumentative essays on task and integration components.

**Component**	**Score awarded**		

	**0**	**1**	**2**
Claim	No claim statement, or an incomprehensible claim	Claim statement presented, but not fully articulated	Claim statement well-articulated
Sources	No source cited	Only one source cited	Multiple sources cited
Justification	No evidence of justifying how the evidence supports the claim	Some evidence of justification for the claim but not fully elaborated	Clear evidence of well-elaborated justification for the claim
Counterargument	No presence of counterargument(s)	Only one counterargument presented, or multiple counterarguments vaguely presented	Multiple counterarguments well-articulated
Writing ability	Incomprehensible paragraphs, or lexical incoherence	Generally comprehensible and moderately coherent lexically	Fully comprehensible, and highly coherent lexically
Critical analysis	No analysis of the sources or information from the sources	Limited analysis of information from sources, or superficial treatment of views or content in the sources	Strong critical analysis of the sources and the views, content, or information from the sources
Synthesis	No evidence of synthesis of information from across the sources	Some evidence of synthesis of information from across the sources in only part of the essay, or in a limited way	Clear evidence of synthesis of information from multiple sources at paragraph level or document level
Overall Cohesion	Disconnected or isolated ideas across paragraphs	Some evidence of connecting ideas within or between paragraphs	Strong connection within and between paragraphs; ideas flow naturally from one to another

Specifically, in terms of adherence to task parameters, effective written products were expected to meet the following criteria. First, the essay should present a clear *claim* reflecting the student's position on the controversial topic. A score of 2 was awarded when there was a clearly identifiable and well-articulated claim statement. A score of 1 was given when the claim was vaguely worded, while a 0 was given when a position statement is absent. Second, the essay included information from multiple *sources* from the digital library. A score of 2 was given when the essay cited at least two sources. A score of 1 was given if only one source was cited, whereas a score of 0 was assigned if no source was referenced.

Third, effective essays presented well-elaborated *justifications* for the claims. A score of 2 was awarded when students presented multiple supporting points for their overarching claims and fully discussed or substantiated those points with examples and evidence (e.g., research findings, data). When students did not fully elaborate or substantiate their supporting arguments, a score of 1 was awarded. Finally, A score of 0 was given when there were no supporting points or details provided or no clear connection between the claim and the supporting details.

Fourth, *counterarguments* representing contrasting or alternative views to students' claims were well articulated and fully addressed. For a score of 2, students needed to present more than one counterpoint to their claims and thoroughly discuss the counterviews or counterevidence. When an essay only briefly mentioned a potential alternative or opposing view without elaboration, a score of 1 was given. A score of 0 was assigned if no counterpoints or counterevidence were addressed.

Finally, a well-crafted essay should manifest adequate *writing ability* that enables idea articulation. A score of 2 was awarded if the essay followed the mechanics of writing and, therefore, was fully comprehensible and coherent at the linguistic level. A score of 1 was given if the writing was comprehensible in general but was only moderately coherent. No credit was awarded if the essay was incomprehensible and incoherent.

As for components of multiple source integration, students' argumentative essays were assessed according to the following criteria. First, *critical analysis* was reflected in students' appraisal of source and content credibility or evaluation of the soundness of the arguments presented in the source documents. Strong critical analysis, warranting a score of 2, was evidenced when students critiqued authors' arguments based on the evidence provided (e.g., identifying that a causal relation cannot be inferred from the correlational data) or when they questioned the trustworthiness of the source to invalidate authors' arguments. In weaker cases of critical analysis that warranted a score of 1, students attempted at analyzing the information from the sources or evaluating authors' views or arguments, but such analyses or evaluations were superficial and unelaborated. Finally, a score of 0 was given if the essay did not demonstrate any evidence of analysis or critique of the sources or their contents.

The second key integration component, *synthesis*, was assessed based on the degree to which students meaningfully consolidated information from multiple sources in making their arguments. In strong cases of synthesis warranting a score of 2, students wove multiple pieces of information from different sources around their arguments, often within several paragraphs or across the entire document. Such synthesis could manifest when students pulled together research findings from two sources that supported the same point, or when they pointed out conflicts between information in two documents. In contrast, weaker evidence of synthesis (warranting a score of 1) was observed when a student included pieces of information from different sources in a loosely connected fashion or only in part of the essay. Further, when no connection between cited sources was identified, a score of 0 was given.

Lastly, *overall cohesion* was evidenced by the degrees of logical connection and flow of ideas within and between paragraphs. In highly cohesive essays (a score of 2), paragraphs were well organized and strongly connected, with clear transitions from one idea to the next, often indicated by connective words and phrases such as “however”, “therefore”, “further”, and “on the contrary.” A score of 1 was awarded if the essay demonstrated weak organizational structure of the ideas across the document or limited flow within or between paragraphs. A point of 0 was given when ideas were presented in a disconnected fashion.

Three independent raters evaluated a randomly selected 10.6% of the essays, yielding an intraclass correlation coefficient (ICC) of 0.93 for interrater reliability on the overall score. The ICCs for individual task parameters were: 0.76 for claim, 0.90 for counterargument, 0.85 for justification, 0.98 for sources, and 0.89 for writing ability. The ICCs for the integration components were: 0.85 for critical analysis, 1.0 for overall cohesion, and 0.85 for synthesis. Prior to scoring, the raters underwent a training process that began with rubric familiarization. They were provided detailed rubrics for each criterion, which explained the scoring scales and what constituted each level of performance. The training then progressed to calibration sessions, where raters scored sample essays together in group settings. During these sessions, they discussed their rationales and worked to resolve any discrepancies, thereby aligning their understanding of the rubrics. After the training, the raters independently scored the essays.

## Data analysis

The data from scoring the task and integration components were used to determine which of the two models more accurately reproduced the argumentative essay writing process with multiple documents using Bayesian network analysis. Each of the eight components that made up the nodes of the Bayesian networks was scored at three levels from 0–2 based on the rubric. Due to insufficient cases of participants who received the lowest score on *writing ability* (*n* = 3), stating a *claim* (*n* = 1), and presenting *justifications* (*n* = 2), the cases were combined with those who received a score of 1. Consequently, these components in the models had only two levels of performance.

Prior to fitting the data to the models, we considered the use of informative priors, which in Bayesian analysis are probability distributions that incorporate existing knowledge about the parameters before observing the data. While previous argumentative writing research exists, it primarily uses non-Bayesian methods making it challenging to translate directly into informative priors. Given this limitation, we opted for weakly informative priors, assigning uniform distribution of students' probabilities of performing at different levels for each component. A uniform distribution, in this context, means that we assigned equal initial probabilities to each performance level, rather than assuming that some levels were more likely than others. Subsequent studies can use information from this research to inform the selection of priors.

After fitting the models to the data, we evaluated how well the predictions made by the models matched the observed data using the leave-one-out cross-validation method. Next, the selected model was used to determine the most crucial components of writing an argumentative essay using multiple documents using the Bayesian Network belief updating procedure.

## Transparency and openness statement

The data used to fit the Bayesian network model is available here https://osf.io/2jh3k/?view_only=d05f1a63bd794f63be960a84e8bd95ee. All other data associated with the study, methods used in the analysis, and materials used to conduct the research will be made available for research purposes upon reasonable request to the corresponding author.

GeNIe Modeller (BayesFusion, LLC) was used for all Bayesian Network modeling described in this study.

## Results and discussion

### Descriptive summary of student performance

Based on the scoring rubric for the argumentative essay, we determined that the mean performance for the eight components was 10.01 (*SD* = 3.41) as presented in Table 3. For components specific to the writing task, the students tended to score between 1 and 2 on the components: writing ability (*M* = 1.44, *SD* = 0.55), claim (*M* = 1.88, *SD* = 0.36), sources (*M* = 1.55, *SD* = 0.76), and justification (*M* = 1.55, *SD* = 0.54). However, a sizeable number of students were unable to provide a counterargument (*M* = 0.90, *SD* = 0.84).

**Table 3:** Descriptive statistics for performance on argumentative essay.

**Components**	**Levels of Performance (*****n*** **=** **105)**						***M (SD)***
	**0**		**1**		**2**		
	***n***	**%**	***n***	**%**	***n***	**%**	
Writing Ability	3	2.86	53	50.48	49	46.67	1.44 (0.55)
Claim	1	0.95	11	10.48	93	88.57	1.88 (0.36)
Sources	17	16.19	13	12.38	75	71.43	1.55 (0.76)
Justification	2	1.90	42	40.00	61	58.10	1.56 (0.54)
Counterargument	42	40.00	31	29.52	32	30.48	0.90 (0.84)
Critical Analysis	59	56.19	23	21.90	23	21.90	0.65 (0.82)
Synthesis	24	22.86	44	41.90	37	35.24	1.12 (0.76)
Overall Cohesion	29	27.62	57	54.29	19	18.10	0.90 (0.67)
Total Score							10.01 (3.41)

This contrast may suggest that these undergraduates were unaware of the role of counterarguments in well-crafted argumentative essay. Alternatively, such a pattern may indicate that these students were operating under the belief that their goal was to “win” an argument and that excluding counterviews would weaken their stance (Brown and Renshaw, 2000; Gilbert, 1997). Relatedly, this frequent absence of counterarguments could reflect myside bias or confirmation bias (Mercier, 2016; Stanovich et al., 2013) in which individuals tend to favor information that confirms their beliefs and disfavor information that counters them.

To gain a more comprehensive understanding of this phenomenon, we conducted a qualitative analysis of the notes from students who performed poorly on the counterargument component. Interestingly, we observed instances where students had engaged with multiple sources during the execution stage, producing high-quality notes, yet failed to incorporate this information into their essays during the production stage. Conversely, students who primarily focused on a single source demonstrated greater proficiency in producing counterarguments. While this qualitative analysis was not the primary focus of our study, it suggests that the struggle with counter argumentation might be partially attributed to the challenges of managing multiple sources of information.

It is also possible that critical analysis of the sources and content is a prerequisite for producing a well-formed counterargument. We tested this possibility in the interconnected Bayesian network model by specifying a causal link from *critical analysis* to *counterargument*. The Bayesian Network model permitted us to test the direction and strength of this causal relation. We observed that students in our sample performed poorly on critical analysis (*M* = 0.65, *SD* = 0.82), which could explain the low performance on the counterargument component.

Students demonstrated superior performance on the synthesis component (*M* = 1.12, *SD* = 0.76) compared to both critical analysis and overall cohesion (*M* = 0.90, *SD* = 0.67) within the integration construct. This relative strength in synthesis may stem from students' experience with MSU assignments in college courses. Additionally, the task design, which instructed students to take notes on selected articles before composing argumentative essays, likely contributed to high synthesis scores. This goal-directed note-taking and review process has been shown to enhance encoding and learning (Kobayashi, 2006), potentially facilitating synthesis. However, the observed discrepancy between synthesis performance and counterargument generation highlights the intricate cognitive processes involved in crafting argumentative essays from multiple documents.

### RQ 1: theoretical models of argumentative essay writing

The interconnected model (Model B in Figure 2) better captured the process of writing an argumentative essay from multiple documents compared to the linear model (Model A in Figure 2). It is worth noting that both models predicted students' performance levels above chance, but the results from the leave-one-out cross-validation procedure indicated that the interconnected model demonstrated higher overall prediction accuracy (65%) than the linear model (63%).

The primary difference between the two models was the interconnections among the various components. In the linear model, enactment of the task components (i.e., source, claim, justification, counterargument) led to synthesis, which gave way to critical analysis and overall cohesion. On the other hand, in the interconnected model, synthesis was a precondition for the successful use of sources, and critical analysis was needed for providing justifications and compelling counterarguments.

The results favoring the interconnected model indicate that a crucial part of the argumentative essay writing process unfolds in the execution stage as outlined by the IF-MT. In this stage, students select credible sources, extract important points and supporting details within and across documents, find associations, and prepare a mental or physical organization of what they have read. Therefore, synthesis and critical analysis appear to be crucial preconditions for enacting the task components in the essay writing process.

It must be noted that while both models demonstrated high prediction accuracy overall, the interconnected model exhibited several advantages in predicting individual task and integration components that we detail below.

#### Interconnected model outperformed linear model in predicting performance on task and integration components

**Figure 4:**
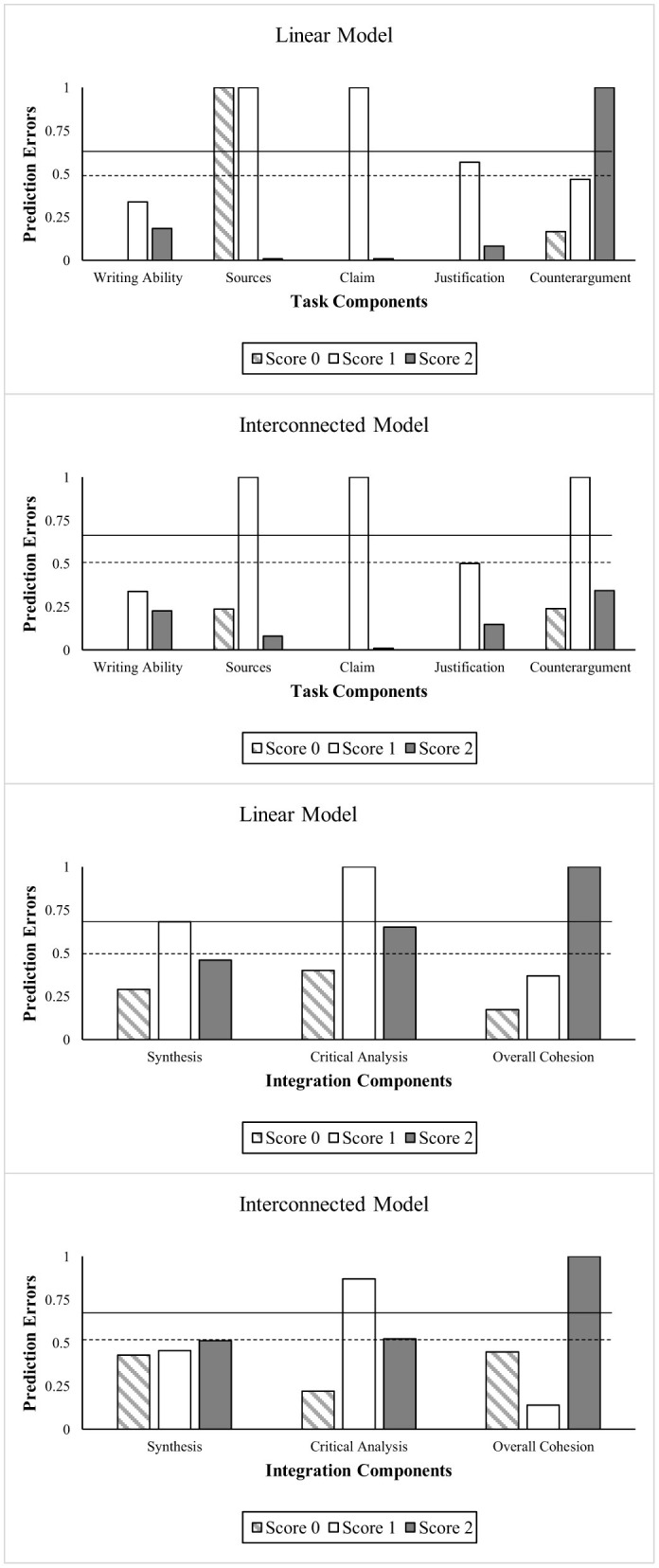
Plots showing prediction errors for task and integration components for linear and interconnected models. The dotted lines represent the prediction error rate by chance. The dotted line at 0.5 is for Writing ability, Claim, and Justification and the solid line at 0.67 is for all other components with three levels of measurement.

The interconnected model correctly predicted the performance of students on task components 72.81% of the time, and its combined prediction accuracy rate for the integration components was 57.78%. In comparison, the linear model was 70.8% correct for task components and 55.23% for the integration components. Although the integration components' prediction accuracies were lower than the task components in both models, they were significantly higher than the prediction accuracy rates by chance (33.3%). Figure 4 presents the prediction error rates for the two models for each of the task and the integration components. The prediction error rate is an inverse of prediction accuracy; so high accuracy and low error indicates a good-fitting model. It is vital to examine the prediction accuracy by task components and the three score levels ranging from 0 to 2 to unpack the granularity of differences between the two models.

Comparing the two models' prediction accuracy for specific task components showed that both performed equally well for the claim and justification components. The primary difference between the models occurred for the components of sources and counterarguments. The linear model could not predict performance on the source component associated with scores 0 and 1. On the other hand, the interconnected model, where synthesis was specified as a precondition for sources, successfully predicted the performance of those scoring 0 and 2 on the source component (lowest and highest scores possible) with an accuracy rate of 76.47 and 92%, respectively.

For counterargument, the linear model predicted performance levels 0 and 1 with accuracy rates of 83.34 and 53.12%, respectively, but could not predict the performance of those at score level 2. On the other hand, the interconnected model accurately predicted the highest performance level 2 (65.62%) and level 0 (76.19%), but struggled with the middle score of 1. The difference between the two models was that the interconnected model specified critical analysis as a precondition for counterargument. It appears that when critical analysis is entered as a prerequisite for counter argumentation, the model performed better at predicting both the lack of counterarguments and exhibiting the highest competence at counter argumentation, but not the intermediate level. This indicates that critical analysis is not associated with only briefly mentioning a counterargument.

Overall, the interconnected model showed high accuracy for each component, but consistently failed to predict performance at score level 1 for claim, sources, justification, and counterargument, with accuracy rates of 0% for most and 50% for justification. Even critical analysis had a low accuracy rate of 13% for level 1. This difficulty in predicting intermediate performance aligns with our earlier observation about counterarguments. It suggests that level 1 performance, which often represents partial or developing skills, has a different relationship with other components than either high (level 2) or low (level 0) performance and the structure of a model fails to capture those relationships.

The interconnected model's struggle with level 1 predictions across components might suggest that the progression from basic to advanced skills in argumentative writing with multiple sources is not straightforward, making intermediate stages particularly challenging to model accurately alongside other performance levels. See Supplementary material for more information comparing the two models.

### RQ 2: relative importance of task and integration components

To address the second research question, we used Bayesian network updating, a method that allows for estimating the probabilities of predefined hypothetical scenarios. Since the interconnected model demonstrated superior performance, we used this model to investigate the relative importance of various components. For instance, in one hypothetical scenario, we forecasted the likelihood of achieving different performance levels across all components when an individual excels in critical analysis. In the Bayesian network framework, this is analogous to probing which components contribute most to achieving the highest level of critical analysis and how this proficiency influences other components connected to it. We carried out this analysis for all task and integration components, synthesizing the results to gain insights.

In the interconnected Bayesian network, we began modeling with the assumption that students had an equal chance of performing at each of the three levels across different components before any observations were made, using what is known as an uninformative prior. This meant that for components with two levels, like writing ability, there was a 50-50 chance for each level, and for components with three levels, like sources, there was an equal chance of being at any of the three levels (33.33%). Next, the student performance data were used to estimate the probability distribution for each component. Finally, we estimated the key task and integration components based on the model using Bayesian network updating procedures.

Components were categorized as crucial to the integration process if achieving high-level performance (i.e., 100% probability of being at level 2) on that component increased the likelihood of performing well (i.e., being at level 2) on other components in the model. In contrast to other statistical tools, Bayesian network updating allowed us to observe the effect of change in performance on one variable on all other variables that serve as a cause or a consequence of that focal variable in modeling a process.

#### Writing ability and source use influenced the process, claim formulation showed no effect

**Figure 5:**
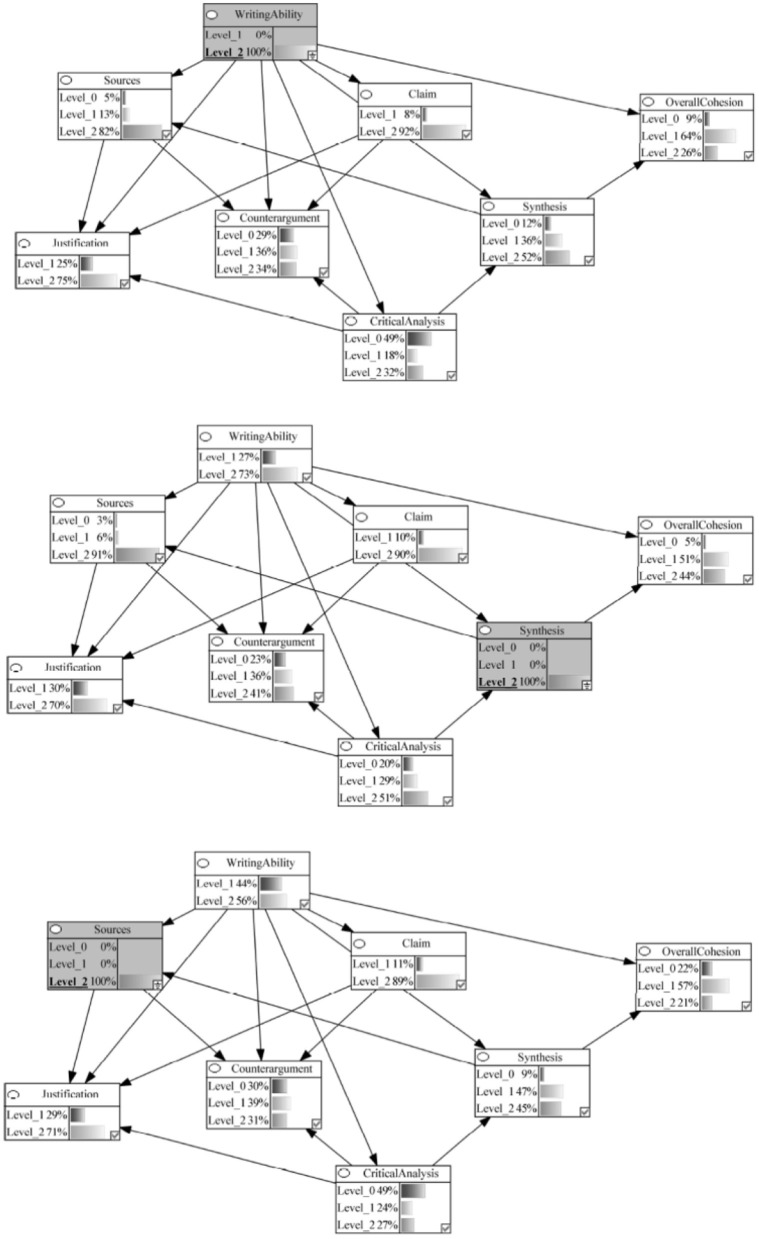
Bayesian networks showing impact of high performance (level 2 probability 100%) on writing ability, synthesis, and sources on the integration process.

In the Bayesian network model, the writing ability component headed the process and was connected to all the remaining task and integration components. Given the written nature of this task, this structure modeled that proficient writing should be a prerequisite skill for manifestation of task (source, claim, justification, and counterargument) and integration (synthesis, critical analysis, and overall cohesion) components that underlie the production of a written argumentative essay. However, our analysis revealed that writing competently (i.e., achieving a score of 2 with 100% probability) directly influenced only one of the task components, justification, and one integration component, synthesis. Specifically, we observed that excelling at the writing ability component increased the probability of also excelling in the corresponding highest levels for justification and synthesis to 75 and 52%, respectively (refer to Figure 5).

Source use strongly impacted the justification component, and source use was itself influenced by synthesis ability. Specifically, the likelihood of achieving a score of 2 on the justification component increased from 55 to 71% when performing with a 100% probability at level 2 on the sources component (see Figure 5). Notably, the integration component synthesis influenced the ability to incorporate multiple sources, a relationship supported by the student performance data. In a hypothetical scenario where synthesis performance was fixed at 100% for score level 2, a corresponding increase was observed in the ability to use multiple sources, rising from a 68% chance to achieve score level 2 to 91% (refer to Figure 5).

**Figure 6:**
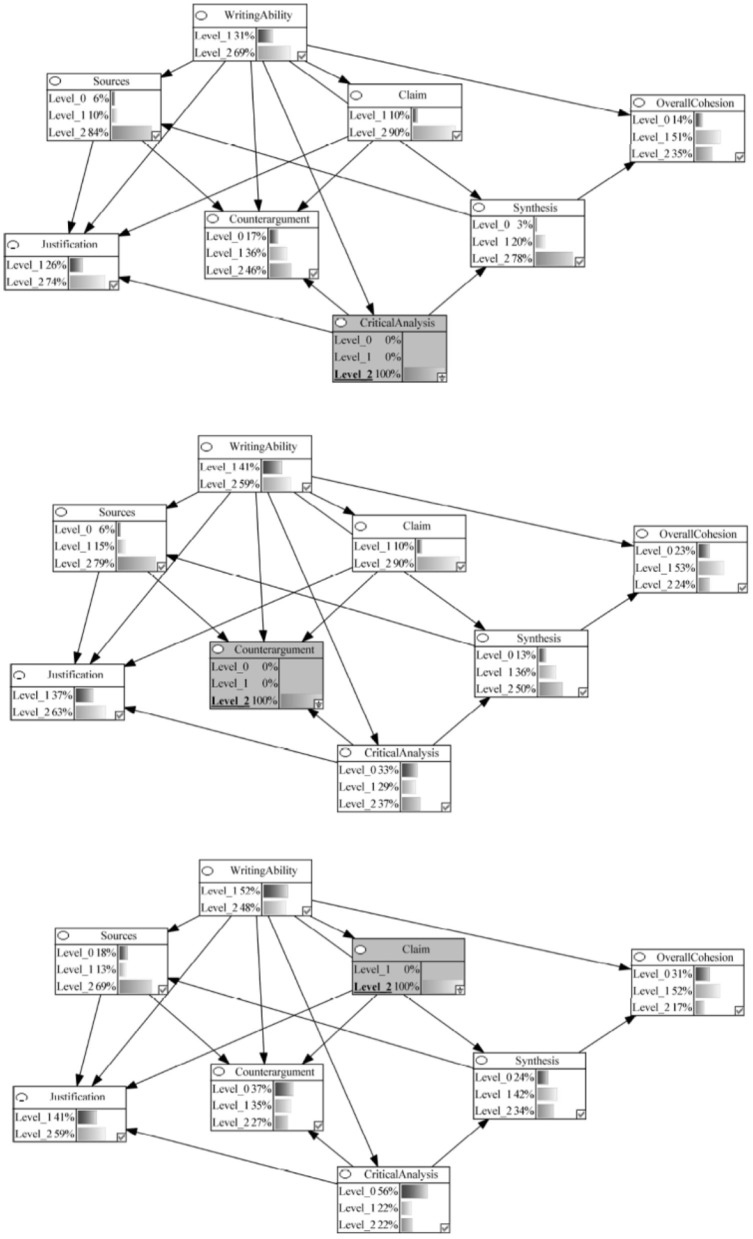
Bayesian networks showing impact of high performance (Level 2 Probability 100%) on critical analysis, counterargument, and claim on the integration process.

The Bayesian updating procedure showed that making a claim was independent of writing ability, and a well-formulated claim was not a prerequisite for sources, justification, or counterargument (see Figure 6). The data from the essays indicated that most undergraduates were able to formulate a well-articulated claim. The controversial topic in this study was selected by a group of undergraduates demographically similar to those who participated in this study. As a result, students appeared to have had an opinion on the topic regardless of the multiple viewpoints presented in the documents. When asked to indicate if their opinion changed due to reading the multiple documents presented in the library, 84% of the students responded, “No, my position has not changed.” This result, therefore, could be an artifact of the fact that most students had an opinion on this topic before they read the topic and simply articulated that opinion in their claim statement. The relations specified by the model should be tested with a topic where students do not have strong preconceived notions.

#### Critical analysis had a substantial impact on task and integration components

The model specified that critical analysis undergirds the ability to present justifications, construct counterarguments, and synthesize information from multiple documents. This pivotal nature of critical analysis was evidenced through Bayesian updating, with the probability of justification increasing to 74%, of counterargument from 27 to 46%, and synthesis from 33 to 78% for being at score level 2 when critical analysis performance was at 100% for the highest score level (see Figure 6).

According to the IF-MT, successful implementation of justification and counterargument components occurs not only at the production stage but begins early on in the process when students analyze the requirements of the argumentative essay task in the preparation stage (see Figure 1). In the argumentative essay, the manifestation of critical analysis appears to be a continuous trace of a critical analytic stance adopted early on during the preparation stage and enacted during execution and production (Sun et al., 2020).

#### Synthesis ability was pivotal to the argumentative writing process

We observed that when students achieved highest points on synthesis (score level 2), the probability of referring to multiple sources in their argumentative essays increased dramatically from 68 to 91%. The Bayesian updating in our network demonstrates that knowing a student has strong synthesis skills allows us to make much more confident predictions about their use of multiple sources in argumentative writing. It is important to note that this pattern also reveals that some students (9%) achieved high synthesis while not referring to multiple sources, and many students (68%) referenced multiple sources without achieving high synthesis scores, underscoring the complexity of the probabilistic relationship between synthesis and multiple source use. Students might successfully synthesize information using fewer sources in some cases, and conversely, students might reference multiple sources without effectively synthesizing the information.

Despite this complexity, our findings align with the Documents Model Framework (Britt and Rouet, 2012; Perfetti et al., 1999) and the IF-MT. According to Documents Model Framework, readers constructing mental representations from multiple documents develop both a situation model (representing document content) and an intertext model (representing source information and connections among documents). Both our empirical results and the theoretical framework establish synthesis as a predictor of students' ability to effectively draw from and reference multiple sources in their writing.

The influence of synthesis extends beyond source use to other aspects of writing quality. For instance, successful synthesis more than doubled the likelihood of producing highly cohesive essays, with overall cohesion rates rising from 17 to 44%. This demonstrates that the cognitive skill of building connections across texts directly translates into measurable improvements in written composition.

Similarly, synthesis showed strong connections to argumentative quality. Previous investigations focusing on synthesis have also attested to the importance of inter-textual relations in forming evidence-backed opinions on controversial topics (e.g., Kobayashi, 2009). In the present study, we found that students can produce strong counterarguments (i.e., achieving a score of 2) when they have at least 50% probability of achieving the highest performance level in synthesis. This is in contrast to the baseline probability of 33% for achieving a score of 2 in synthesis, which represents the chance probability if scores were randomly distributed across the three possible levels (0, 1, and 2) with equal likelihood (refer to Figure 6).

Synthesis, which is the opposite of piecemealing, and a manifestation of the ability to draw intra- and inter-textual connections in documents emerged as an indispensable competency to support integration in the MSU argumentative essay task.

## Conclusion and implications

The aims of this study were multifaceted. We set out to model the process of written argumentation using Bayesian network analysis in the context of a multiple source use task. We tested the comparative prediction accuracy of two theoretically viable models—the linear model and the interconnected model. The models were constructed based on argumentation and multiple source use literatures. Although both models made better than chance predictions, the interconnected model reproduced the data with higher accuracy than the linear model. After selecting the higher-performing model, we used Bayesian updating as an innovative method to pinpoint the key components in the argumentative writing process in MSU contexts. The insights gleaned from this analysis can be used to inform instruction of argumentative writing in the internet age, where students have to contend with an informational deluge.

## Implications for research: modeling the componential process of argumentative essay writing

This study is among the first attempts to model the process of writing an argumentative essay using Bayesian network analysis. Bayesian network analysis is distinct from other modeling tools because it provides information on student performance in probabilistic terms and allows for testing causal connections. The evidence supporting the interconnected model indicated that the integration components, especially critical analysis, are a key driver of the argumentative writing process. This finding lends credence to the stage-based framework (IF-MT) proposed by List and Alexander (2019), wherein essential cognitive actions are undertaken well before the production of the essay. They forward that *preparation* and *execution* are crucial stages before *production*. It is in these earlier two stages that we see the enactment of task analysis, building of intra- and inter-textual links, and critical analysis of the sources and the content contained in them. But the manifestation of these earlier actions is readily available in the *production* stage, where students actually produce the written essay. The strength of the modeling procedure used in this study is that we were able to gain insights into causal links among the components that undergird the process of writing by assessing the product.

The Bayesian network modeling approach we presented in this study can be flexibly adapted to model other MSU writing tasks. The models we tested were rooted in the production phase of the IF-MT and incorporated both task-specific and general integration components. The hybrid Bayesian network modeling approach, blending theory with tasks with computational learning, provides a framework for researchers and practitioners to investigate other writing task processes. One possible avenue for future MSU investigations would be to retain the integration components while adjusting the task parameters to suit other types of writing tasks.

Bayesian analysis becomes even more powerful as we gather additional information and build on previous investigations. In this study, we did not have any prior information on the performance of students. However, now we have data about performance on each component, for example, we know that most undergraduates can provide a claim statement but struggle with counterarguments. Future research studies can use more informative priors by drawing from the current research to improve the predictive power of Bayesian networks. In the Supplementary material, we provide complete conditional probability tables for each node in our network, which researchers can directly incorporate as Dirichlet priors when studying similar populations. For educational practice and intervention research, we can design instruction that supports the needs of specific types of students with increasing precision by collecting more data.

## Implications for practice: supporting integration in an MSU argumentative essay task

Our study on undergraduate argumentative essay writing using multiple documents revealed several key components that students struggle with. Critical analysis emerged as a crucial element, with its causal connection to counterargument playing a pivotal role in improving the overall integration process. We focused on these aspects because they underlie effective decision-making, problem-solving, and functioning in democratic societies, as noted by scholars like Dewey (1933) and Diamond (2013). Additionally, the ability to synthesize information surfaced as another critical parameter.

The findings indicated that while undergraduates can provide justifications given adequate writing skills, their major weakness lies in considering and rebutting counter views. This shortcoming is significant because constructing a reasoned argument requires more than mere justifications; it demands the consideration of multiple perspectives and the evaluation of evidence supporting contrasting views. To address this, we propose that interventions or instruction supporting counter argumentation should focus on honing students' critical analysis skills. One avenue for doing this would be through training in relational reasoning.

Relational reasoning, the ability to discern patterns in information streams, encompasses four distinct forms: analogical, anomalous, antinomous, and antithetical (see Table 4). By developing these skills, students could draw deeper connections among multiple documents, identifying similarities, dissimilarities, and contradictions across texts. This enhanced ability would prepare them to critically analyze complex information, synthesize ideas from various sources, and develop counterarguments, ultimately improving their overall argumentation skills.

**Table 4:** Forms of relational reasoning.

**Form**	**Definition**	**Example in argumentation context**
Analogy	Recognizing meaningful similarities	Identifying when two sources present parallel arguments
Anomaly	Identifying deviations from patterns	Recognizing when evidence contradicts an established pattern
Antinomy	Recognizing mutual exclusivity	Understanding when accepting one position necessitates rejecting another
Antithesis	Identifying direct oppositions	Recognizing when sources directly contradict each other

Our study methodology, which required students to compose essays using only their notes without direct access to digital texts, potentially encouraged deeper engagement with source materials during the initial reading phase. This approach may have led to more thorough note-taking and enhanced synthesis scores. However, the significant discrepancy observed between synthesis and counter argumentation performance suggests that even with this potentially beneficial note-taking process, students still struggled with more complex argumentative task components.

In real-life settings, where students would likely have access to original texts, we might expect some differences in performance. While direct text referencing might improve accuracy, it could potentially reduce the depth of engagement that our task structure encouraged. Educators applying our findings should consider incorporating strategies that combine the benefits of note-taking with the practical reality of text availability. This could include teaching effective annotation and quick-reference techniques, ensuring that students develop both deep engagement with texts and practical skills for managing multiple sources in their argumentative writing.

## Limitations and future directions

A primary limitation of the current study is the use of a single task topic and one essay per student, which potentially restricts the external validity of our findings. Additionally, to reflect the three stages of the IF-MT framework, we directed students to first make notes and then use only those notes to craft their essays without referring back to the original sources or revising their work. While this controlled approach aligned with our theoretical framework and may have enhanced synthesis skills, it deviated from authentic writing practices where students typically consult source materials throughout the writing process and revise their essays. Future studies should consider incorporating a more diverse range of topics, allow students to consult articles during writing, and provide opportunities for revision to better represent how argumentative writing naturally unfolds in academic contexts.

While our study design prioritized ecological validity by allowing students to complete the assignment as homework, this approach introduced a tradeoff with experimental control and may have contributed to variability that a controlled laboratory setting could have minimized. Despite this limitation, the homework format better reflects the conditions under which students typically complete writing assignments. The compromises between naturalistic conditions and procedural control that we made highlight the complex challenges in studying writing processes in classroom settings.

Turning to the central aspect of our study, a key limitation of our modeling approach was that we only compared two alternative Bayesian network models. While our methodology and task instructions (e.g., requiring students to forward a claim and use only their notes to write essays) necessarily imposed certain constraints on component sequences, additional plausible models exist that we did not test. For instance, models that modify individual connections between components in our Models A and B, or models incorporating direct causal influences on claim formulation, might offer alternative explanations of the data. The *apriori* models we tested were theoretically driven, based on the literature on argumentation and multiple source use. However, we acknowledge that argumentative writing involves complex processes that could be represented through various network structures. For instance, we recognize the complexity in relations between specific components, such as synthesis and multiple source use, where alternative causal directions may be plausible—it could be that exposure to multiple sources enables better synthesis, rather than synthesis driving multiple source use, or that students might successfully synthesize with fewer sources while others might reference multiple sources without effectively synthesizing the information. Future research should systematically test a broader range of alternative models, including those with different directional relationships between components, to strengthen causal inferences about argumentative writing processes.

Another limitation of our approach is that it does not fully capture the entire writing process as laid out by the IF-MT. Specifically, the Preparation and Execution stages were not incorporated into the Bayesian network analysis. Our working assumption was that we would see traces of the processes that occur during the previous stages in the written essay (Production stage). However, we found as part of unplanned analysis that there was a disconnect in the quality of the notes and the quality of the essays. Future research should aim to include these crucial stages to provide a more comprehensive understanding of the writing process.

The IF-MT, drawing from the cognitive affective engagement model (CAEM; List and Alexander, 2017), articulates that students can adopt four stances toward task completion—disengaged, affectively engaged, evaluative, critical analytic. According to the IF-MT, all four of these stances combine affective and cognitive aspects that are not just preexisting individual attributes but also iterate within the context of the task. It follows that if educators are interested in developing critical analytic orientation in students, instruction should consider the role of affective dimensions such as engagement in and motivation for the task in addition to developing cognitive resources. The current study does not measure affective involvement. However, we concede that this dimension also plays a role in successfully integrating multiple documents in a goal-directed context. Further, it is essential to note that epistemic beliefs are also significant predictors of the processing of multiple documents (Bråten et al., 2011; Ferguson et al., 2013), but we did not include them in our Bayesian model. Future investigations should study the distinct contribution of affect and epistemic beliefs in integration and how they undergird the higher-order cognitive skills, such as, critical analysis.

## Final word

The 21^st^-century context is riddled with open-ended, ill-structured problems in information-rich digital spaces. One of the pressing challenges that educational researchers must respond to is how to foster the habits of mind that equip students to integrate relevant information from multiple sources to solve a problem. In this investigation, we have demonstrated an innovative, adaptive, and theoretically driven method for modeling a written task using multiple documents of variable source and content credibility. Using this method, we not only shed light on how the componential process unfolds but also determined vital areas where students require support. Subsequent researchers using Bayesian analysis should build on the findings of this study to further enhance our understanding of the process of writing in MSU contexts and the challenges that students face.

## Data Availability

The raw data supporting the conclusions of this article will be made available by the authors, without undue reservation.
