# Supplementary material for: Componential modeling of argumentative essay writing from multiple online sources: a Bayesian network approach
Source: Front Psychol. 2025 May 15;16:1560088. doi: 10.3389/fpsyg.2025.1560088 (PMC12119471; doi:10.3389/fpsyg.2025.1560088)
Supplement: Supplementary file 1 [file Supplementary_file_1.docx]

Supplementary Material to “Componential Modeling of Argumentative Essay Writing from Multiple Online Sources: A Bayesian Network Approach”

Supplemental Material

**Mathematical Representation of a Bayesian Network**

The joint distribution for a Bayesian network is given by:

$P\left( x_{1},\ldots x_{n} \right) =\prod_{i} P\left( x_{i} | pa_{i} \right)$ (S1)

Hence, for our Bayesian network with eight variables (components) and the specific interconnections modeling integration in the production stage of the IF-MT, the mathematical representation of the join probability distribution is given by:

$$P\left( x_{1},x_{2},x_{3},x_{4},x_{5},x_{6},x_{7},x_{8} \right)=P\left( x_{1} \right)P\left( x_{2} | x_{1} \right)P\left( x_{3} | x_{1},x_{7} \right)P\left( x_{4} | x_{1},x_{2},x_{3},x_{6} \right)P\left( x_{5} | x_{1},x_{2},x_{3},x_{6} \right)$$

$P\left( x_{6} | x_{1} \right)P\left( x_{7} | x_{1},x_{6} \right)P\left( x_{8} | x_{1},x_{7} \right)$ (S2)

Where,

*x_1_* is *Writing Ability*

*x_2_* is *Claim*

*x_3_* is *Sources*

*x_4_* is *Justification*

*x_5_* is *Counterargument*

*x_6_* is *Critical Analysis*

*x_7_* is *Synthesis*

*x_8_* is *Overall Cohesion*

Equation (S2) is represented in the form of a Directed Acyclical Graph (DAG) in the body of the paper (see Figure 2). The equation shows in mathematical form how the dependencies encoded in the graphical representation of the Bayesian network used to model integration process in this study map into the probability space through conditional (in)dependence relationships.

**Model Checking: Confusion Matrices and ROC Curve**

The confusion matrices compare predicted with actual outcomes, thereby, providing information about the accuracy of model predictions (Pearson, 1904; Ting, 2017). Table S1 presents the confusion matrices for each of the components in our model.

Further, the quality of the model can be graphically evidenced through Receiver Operating Characteristic (ROC) curves for each of the states of the components. An ROC curve is a plot showing the relation between true positive rate on the y-axis (i.e., sensitivity) and false positive rate on the x-axis (i.e., 1 – specificity). In the ROC, the 45-degree line represents a random guess, hence, if the curve falls above the line, it is indicative of a model with high accuracy and vice-versa.

The ROC curves for level 2 (highest level of performance) of the components of the Bayesian network model are presented below in Figure S1 and S2. As shown in the figures the components *writing ability, sources, justification, counterargument, critical analysis, synthesis, overall cohesion* were predicted with acceptable accuracy at level 2. We present the ROC curves only for level 2 because we were primarily interested in the accuracy of the model in predicting successful integration in the context of an argumentative essay task.

**Table S1**

*Confusion Matrices Depicting Predicted vs. Actual Outcomes for the Linear Model*

Writing Ability

| Actual | Predicted | |
| --- | --- | --- |
|  | Level 1 | Level 2 |
| Level 1 | **37** | 19 |
| Level 2 | 9 | **40** |

Claim

| Actual | Predicted | |
| --- | --- | --- |
|  | Level 1 | Level 2 |
| Level 1 | **0** | 12 |
| Level 2 | 0 | **93** |

Sources

| Actual | Predicted | |  |
| --- | --- | --- | --- |
|  | Level 0 | Level 1 | Level 2 |
| Level 0 | **0** | 0 | 17 |
| Level 1 | 0 | **0** | 13 |
| Level 2 | 0 | 0 | **75** |

Justification

| Actual | Predicted | |
| --- | --- | --- |
|  | Level 1 | Level 2 |
| Level 1 | **19** | 25 |
| Level 2 | 5 | **56** |

Counterargument

| Actual | Predicted | |  |
| --- | --- | --- | --- |
|  | Level 0 | Level 1 | Level 2 |
| Level 0 | **35** | 2 | 5 |
| Level 1 | 1 | **17** | 13 |
| Level 2 | 15 | 17 | **0** |

*Note.* The predicted values are bolded.

Critical Analysis

| Actual | Predicted | |  |
| --- | --- | --- | --- |
|  | Level 0 | Level 1 | Level |
| Level 0 | **56** | 0 | 3 |
| Level 1 | 19 | **0** | 4 |
| Level 2 | 15 | 0 | **8** |

Synthesis

| Actual | Predicted | |  |
| --- | --- | --- | --- |
|  | Level 0 | Level 1 | Level 2 |
| Level 0 | **17** | 9 | 0 |
| Level 1 | 1 | **14** | 29 |
| Level 2 | 5 | 12 | **20** |

Overall Cohesion

| Actual | Predicted | |  |
| --- | --- | --- | --- |
|  | Level 0 | Level 1 | Level 2 |
| Level 0 | **24** | 1 | 4 |
| Level 1 | 21 | **36** | 0 |
| Level 2 | 5 | 14 | **0** |

**Table S2**

*Confusion Matrices Depicting Predicted vs. Actual Outcomes for the Interconnected Model*

Writing Ability

| Actual | Predicted | |
| --- | --- | --- |
|  | Level 1 | Level 2 |
| Level 1 | **37** | 19 |
| Level 2 | 11 | **38** |

Claim

| Actual | Predicted | |
| --- | --- | --- |
|  | Level 1 | Level 2 |
| Level 1 | **0** | 12 |
| Level 2 | 0 | **93** |

Sources

| Actual | Predicted | |  |
| --- | --- | --- | --- |
|  | Level 1 | Level 2 | Level 3 |
| Level 0 | **13** | 0 | 4 |
| Level 1 | 5 | **0** | 8 |
| Level 2 | 6 | 0 | **69** |

Justification

| Actual | Predicted | |
| --- | --- | --- |
|  | Level 1 | Level 2 |
| Level 1 | **22** | 22 |
| Level 2 | 9 | **52** |

Counterargument

| Actual | Predicted | |  |
| --- | --- | --- | --- |
|  | Level 1 | Level 2 | Level 3 |
| Level 0 | **35** | 0 | 7 |
| Level 1 | 21 | **0** | 10 |
| Level 2 | 11 | 0 | **21** |

*Note.* The predicted values are bolded.

Critical Analysis

| Actual | Predicted | |  |
| --- | --- | --- | --- |
|  | Level 1 | Level 2 | Level 3 |
| Level 0 | **45** | 8 | 6 |
| Level 1 | 14 | **3** | 6 |
| Level 2 | 15 | 0 | **8** |

Synthesis

| Actual | Predicted | |  |
| --- | --- | --- | --- |
|  | Level 1 | Level 2 | Level 3 |
| Level 0 | **15** | 7 | 2 |
| Level 1 | 5 | **22** | 17 |
| Level 2 | 2 | 17 | **18** |

Overall Cohesion

| Actual | Predicted | |  |
| --- | --- | --- | --- |
|  | Level 1 | Level 2 | Level 3 |
| Level 0 | **14** | 15 | 0 |
| Level 1 | 9 | **48** | 0 |
| Level 2 | 1 | 18 | **0** |

**Figure S1**

*ROC Curves Representing Linear Model’s Prediction Accuracy for Task Components at Level 2*

**Writing Ability Claim**

**
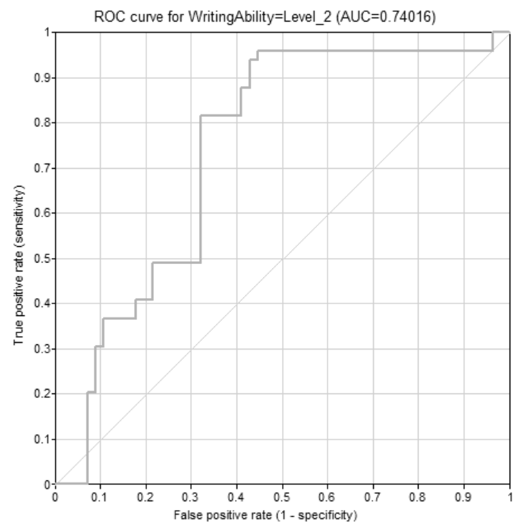

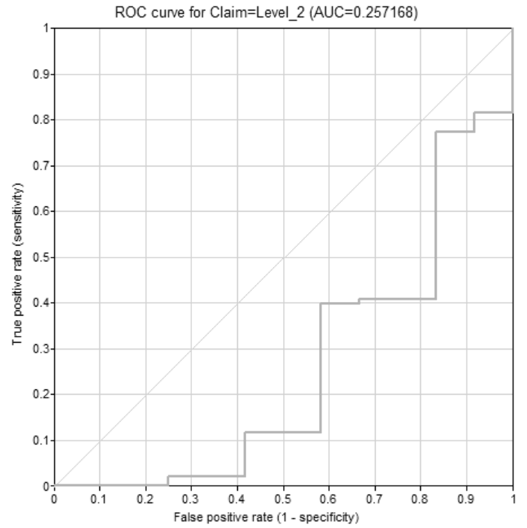
**

**
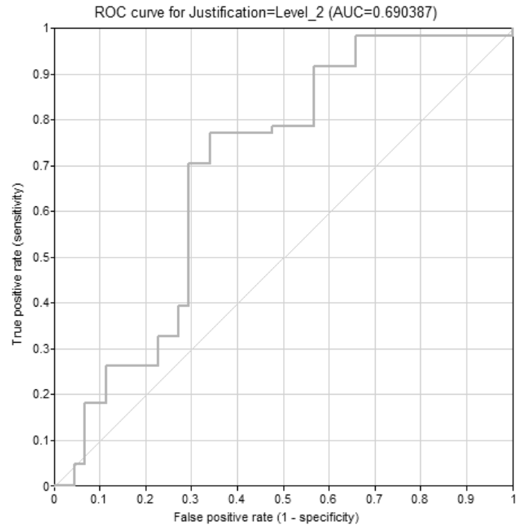

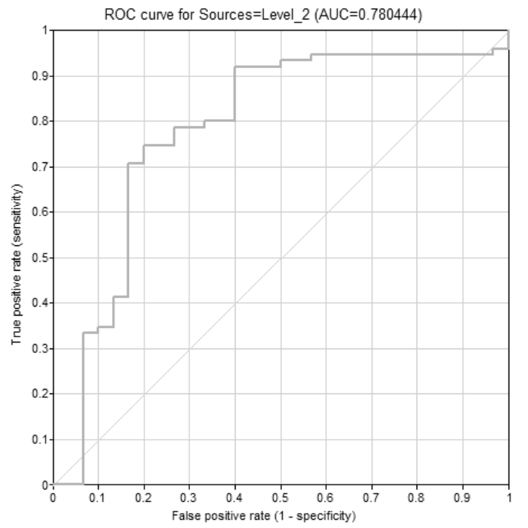
 Sources Justification**

**Counterargument**

**
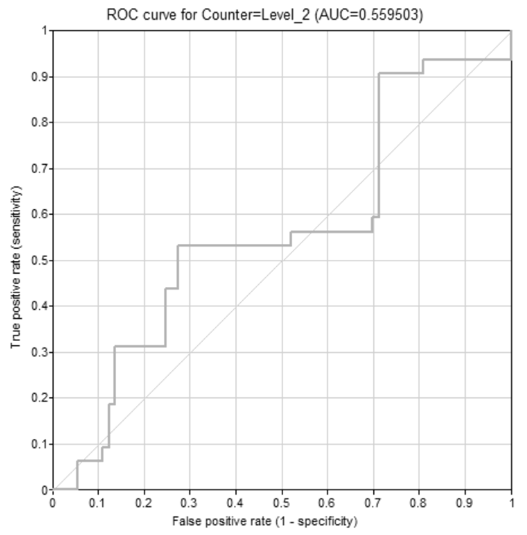
**

*Note.* AUC = Area Under Curve; values greater than 0.5 indicate higher accuracy of prediction

**Figure S2**

*ROC Curves Representing Linear Model’s Prediction Accuracy for Integration Components at Level 2*

**
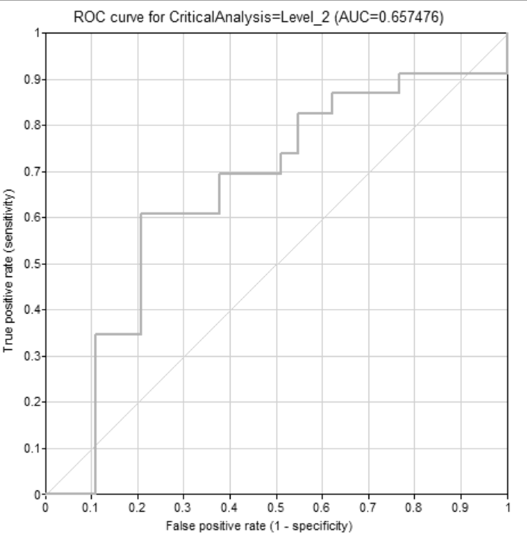
Synthesis Critical Analysis**

*
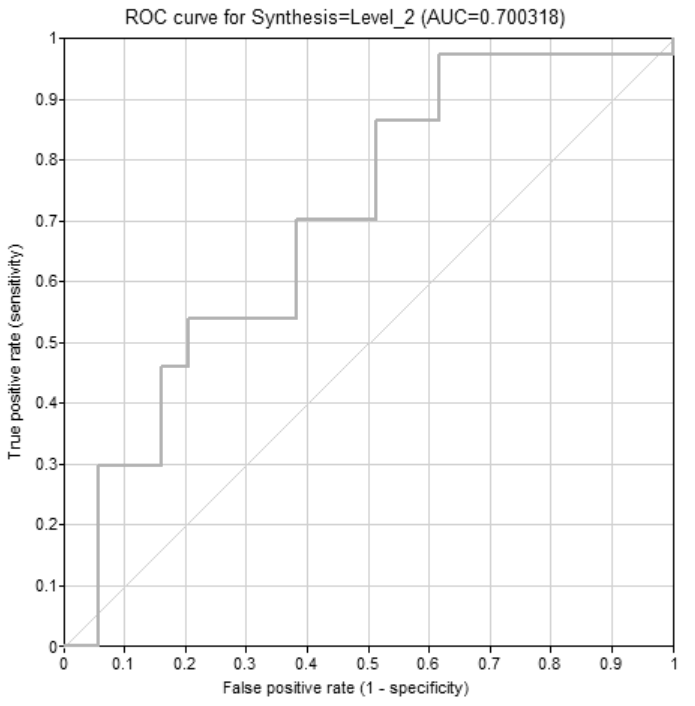
*

**Overall Cohesion***
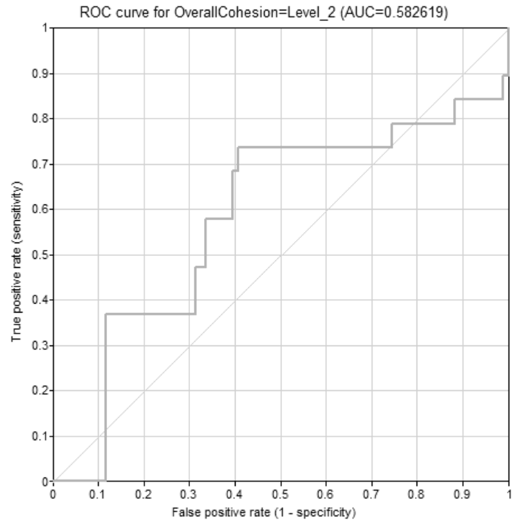
*

*Note.* AUC = Area Under Curve; values greater than 0.5 indicate higher accuracy of prediction

**Figure S3**

*ROC Curves Representing Interconnected Model’s Prediction Accuracy for Task Components at Level 2*

**
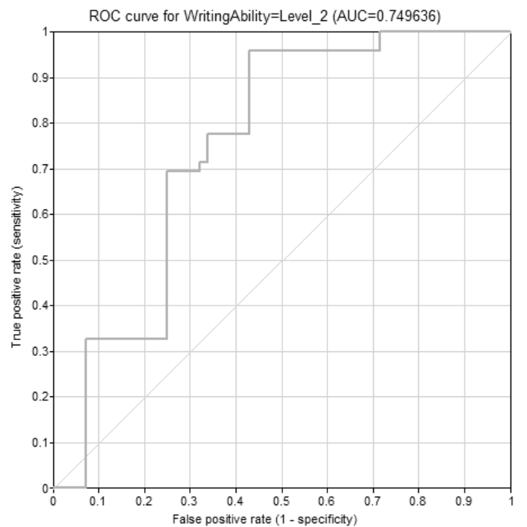

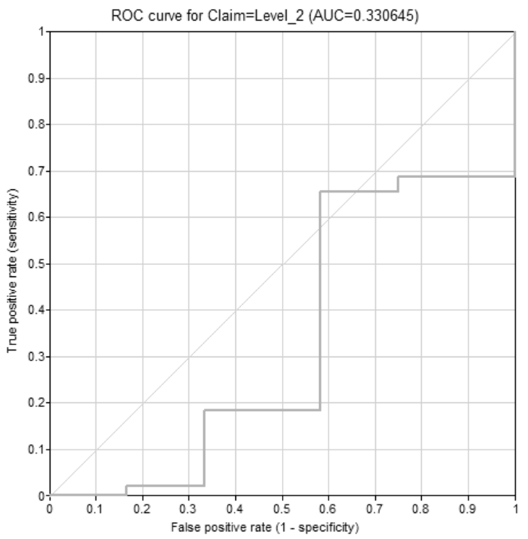
Writing Ability Claim**

**
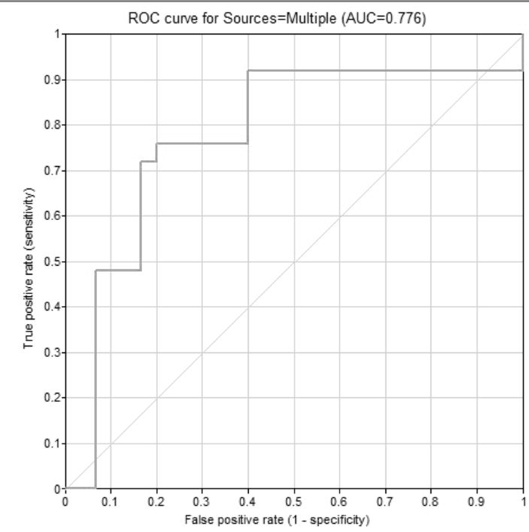

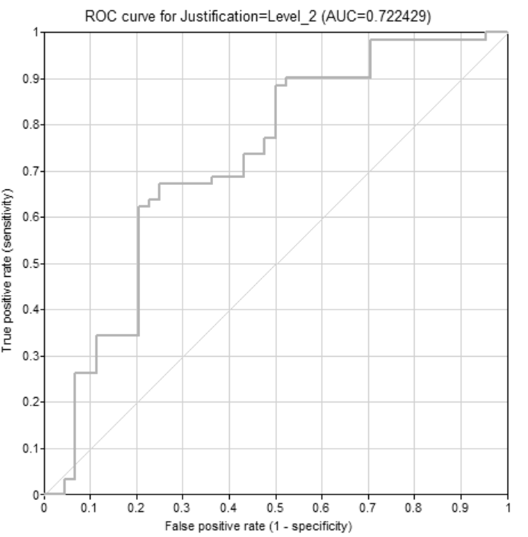
 Sources Justification**

**
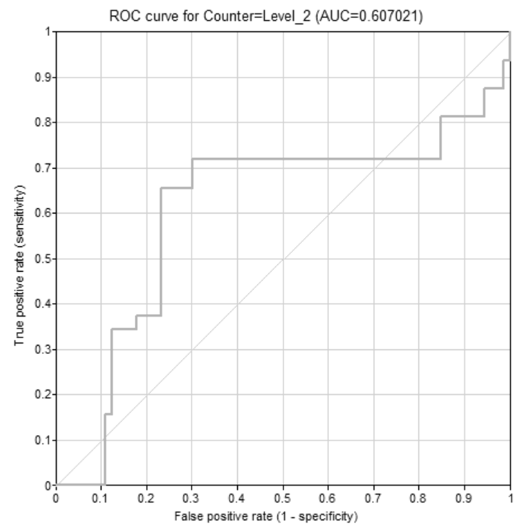
 Counterargument**

*Note.* AUC = Area Under Curve; values greater than 0.5 indicate higher accuracy of prediction

**Figure S4**

*ROC Curves Representing Interconnected Model’s Prediction Accuracy for Integration Components at Level 2*

**Synthesis Critical Analysis**


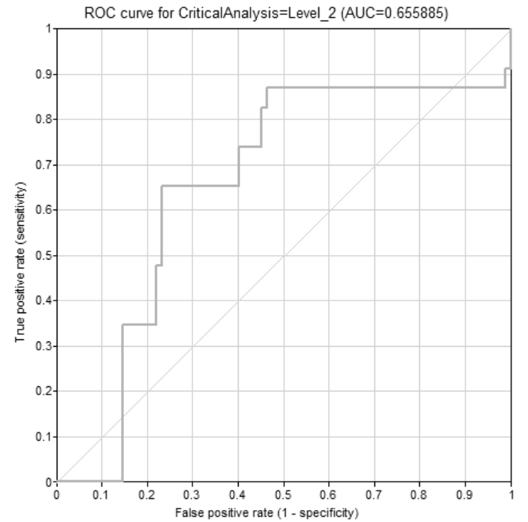

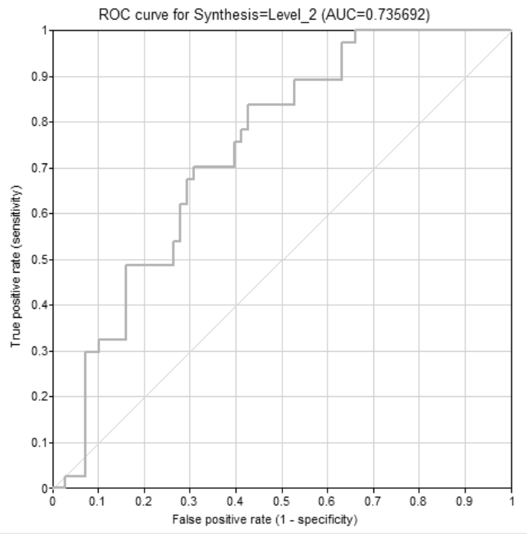


**Overall Cohesion**

**
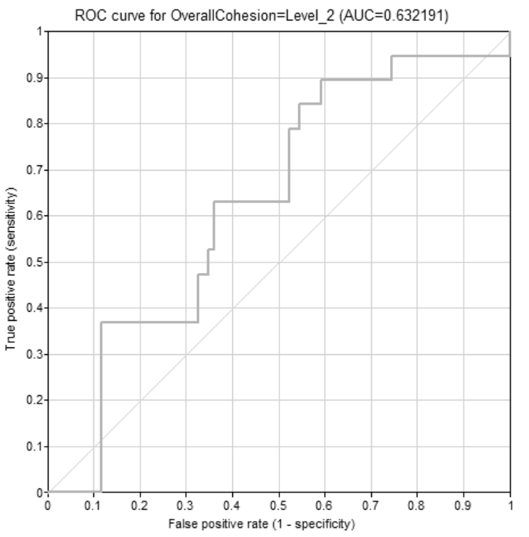
**

*Note.* AUC = Area Under Curve; values greater than 0.5 indicate higher accuracy of prediction.

**Bayesian Updating or Inference**

***Conditional Probability Tables***

The conditional probability tables provide the estimation of the task and integration components. They are the core of Bayesian network underlying each node of the network. In the case of the Bayesian network used in this study, each node represents one of the eight components (five task and three integration components). *Writing ability* was the only parentless node in the network, therefore, it has an unconditional probability table associated with it. For all of the components, the assumption (prior) reflected the belief that students are equally likely to perform at any of the three levels. The conditional probability tables for each of the task components are given in Table S2 and for the integration components in Table S3. The first column lists the parents of the node, that is, the components that are prerequisite for the enactment of the component in question.

**Table S3**

| Justification | | | | | | | | | | | | | | | | | | | | | | | | | | | | | | | | | | | | |
| --- | --- | --- | --- | --- | --- | --- | --- | --- | --- | --- | --- | --- | --- | --- | --- | --- | --- | --- | --- | --- | --- | --- | --- | --- | --- | --- | --- | --- | --- | --- | --- | --- | --- | --- | --- | --- |
| WA | L1 | | | | | | | | | | | | | | | | | | L2 | | | | | | | | | | | | | | | | | |
| So | L1 | | | | | | L2 | | | | | | L3 | | | | | | L1 | | | | | | L2 | | | | | | L3 | | | | | |
| CA | L1 | | L2 | | L3 | | L1 | | L2 | | L3 | | L1 | | L2 | | L3 | | L1 | | L2 | | L3 | | L1 | | L2 | | L3 | | L1 | | L2 | | L3 | |
| Cl | L1 | L2 | L1 | L2 | L1 | L2 | L1 | L2 | L1 | L2 | L1 | L2 | L1 | L2 | L1 | L2 | L1 | L2 | L1 | L2 | L1 | L2 | L1 | L2 | L1 | L2 | L1 | L2 | L1 | L2 | L1 | L2 | L1 | L2 | L1 | L2 |
| L1 | 0.9 | 1.0 | 0.8 | 0.9 | 0.8 | 0.9 | 0.8 | 0.6 | 0.9 | 0.8 | 0.8 | 0.8 | 1.0 | 0.3 | 0.4 | 0.6 | 0.9 | 0.3 | 0.8 | 0.4 | 0.8 | 0.8 | 0.8 | 0.8 | 0.8 | 0.6 | 0.8 | 0.8 | 0.8 | 0.6 | 0.6 | 0.1 | 0.8 | 0.4 | 0.6 | 0.1 |
| L2 | 0.1 | 0.0 | 0.3 | 0.1 | 0.3 | 0.1 | 0.3 | 0.4 | 0.1 | 0.3 | 0.3 | 0.3 | 0.1 | 0.7 | 0.6 | 0.4 | 0.1 | 0.7 | 0.3 | 0.6 | 0.3 | 0.3 | 0.3 | 0.3 | 0.3 | 0.5 | 0.3 | 0.3 | 0.3 | 0.4 | 0.4 | 0.9 | 0.3 | 0.6 | 0.4 | 0.9 |

| Counterargument | | | | | | | | | | | | | | | | | | | | | | | | | | | | | | | | | | | | |
| --- | --- | --- | --- | --- | --- | --- | --- | --- | --- | --- | --- | --- | --- | --- | --- | --- | --- | --- | --- | --- | --- | --- | --- | --- | --- | --- | --- | --- | --- | --- | --- | --- | --- | --- | --- | --- |
| WA | L1 | | | | | | | | | | | | | | | | | | L2 | | | | | | | | | | | | | | | | | |
| So | L1 | | | | | | L2 | | | | | | L3 | | | | | | L1 | | | | | | L2 | | | | | | L3 | | | | | |
| CA | L1 | | L2 | | L3 | | L1 | | L2 | | L3 | | L1 | | L2 | | L3 | | L1 | | L2 | | L3 | | L1 | | L2 | | L3 | | L1 | | L2 | | L3 | |
| Cl | L1 | L2 | L1 | L2 | L1 | L2 | L1 | L2 | L1 | L2 | L1 | L2 | L1 | L2 | L1 | L2 | L1 | L2 | L1 | L2 | L1 | L2 | L1 | L2 | L1 | L2 | L1 | L2 | L1 | L2 | L1 | L2 | L1 | L2 | L1 | L2 |
| L0 | 0.1 | 0.6 | 0.2 | 0.6 | 0.2 | 0.6 | 0.2 | 0.4 | 0.6 | 0.6 | 0.2 | 0.2 | 0.4 | 0.4 | 0.1 | 0.3 | 0.1 | 0.2 | 0.2 | 0.6 | 0.2 | 0.2 | 0.2 | 0.2 | 0.2 | 0.6 | 0.2 | 0.2 | 0.2 | 0.1 | 0.1 | 0.4 | 0.2 | 0.2 | 0.1 | 0.2 |
| L1 | 0.8 | 0.3 | 0.7 | 0.3 | 0.7 | 0.3 | 0.7 | 0.2 | 0.3 | 0.1 | 0.7 | 0.7 | 0.3 | 0.4 | 0.3 | 0.5 | 0.8 | 0.3 | 0.7 | 0.3 | 0.7 | 0.7 | 0.7 | 0.7 | 0.7 | 0.1 | 0.7 | 0.7 | 0.7 | 0.2 | 0.9 | 0.4 | 0.7 | 0.2 | 0.2 | 0.4 |
| L2 | 0.1 | 0.1 | 0.2 | 0.1 | 0.2 | 0.1 | 0.2 | 0.4 | 0.1 | 0.2 | 0.2 | 0.2 | 0.2 | 0.1 | 0.6 | 0.3 | 0.1 | 0.5 | 0.2 | 0.1 | 0.2 | 0.2 | 0.2 | 0.2 | 0.2 | 0.2 | 0.2 | 0.2 | 0.2 | 0.7 | 0.1 | 0.2 | 0.2 | 0.6 | 0.7 | 0.5 |

*Conditional Probability Tables Underlying the Estimation of the Task Components in the Interconnected Model (Best-Fitting Model)*

| Writing Ability | |
| --- | --- |
| No Parent Node |  |
| L1 | 0.53 |
| L2 | 0.47 |

| Claim | | |
| --- | --- | --- |
| WA | L1 | L2 |
| L1 | 0.14 | 0.08 |
| L2 | 0.86 | 0.91 |

| Sources | | | | | | |
| --- | --- | --- | --- | --- | --- | --- |
| WA | L1 | | | L2 | | |
| Sy | L0 | L1 | L2 | L0 | L1 | L2 |
| L0 | 0.63 | 0.16 | 0.05 | 0.26 | 0.03 | 0.02 |
| L1 | 0.11 | 0.18 | 0.02 | 0.53 | 0.07 | 0.07 |
| L2 | 0.26 | 0.65 | 0.93 | 0.21 | 0.90 | 0.90 |

*Note.* CA = Critical Analysis; Cl = Claim; So = Sources; Sy = Synthesis; WA = Writing Ability; L0 = Level 0; L1 = Level 1; L2 = Level 2.

**Table S4**

| Synthesis | | | | | | |
| --- | --- | --- | --- | --- | --- | --- |
| WA | L1 | | | L2 | | |
| CA | L0 | L1 | L2 | L0 | L1 | L2 |
| L0 | 0.45 | 0.22 | 0.04 | 0.21 | 0.04 | 0.02 |
| L1 | 0.48 | 0.43 | 0.56 | 0.58 | 0.35 | 0.03 |
| L2 | 0.06 | 0.34 | 0.39 | 0.20 | 0.61 | 0.94 |

*Conditional Probability Tables Underlying the Estimation of the Integration Components in the Interconnected Model (Best-Fitting Model)*

| Critical Analysis | | |
| --- | --- | --- |
| WA | L1 | L2 |
| L0 | 0.62 | 0.49 |
| L1 | 0.25 | 0.18 |
| L2 | 0.13 | 0.32 |

| Overall Cohesion | | | | | | |
| --- | --- | --- | --- | --- | --- | --- |
| WA | L1 | | | L2 | | |
| Syn | L0 | L1 | L2 | L0 | L1 | L2 |
| L0 | 0.69 | 0.53 | 0.08 | 0.48 | 0.05 | 0.03 |
| L1 | 0.25 | 0.47 | 0.55 | 0.51 | 0.89 | 0.50 |
| L2 | 0.05 | 0.00 | 0.37 | 0.01 | 0.06 | 0.47 |

*Note.* L0 = Level 0; L1 = Level 1; L2 = Level 2.

*Inference Algorithm*

We used clustering algorithm for belief updating, which is the default algorithm in GeNIe (Lauritzen and Spiegelhalter, 1988).
